# Genome-wide association study identifies a major gene for beech bark disease resistance in American beech (*Fagus grandifolia* Ehrh.)

**DOI:** 10.1186/s12864-017-3931-z

**Published:** 2017-07-20

**Authors:** Irina Ćalić, Jennifer Koch, David Carey, Charles Addo-Quaye, John E. Carlson, David B. Neale

**Affiliations:** 1 Department of Plant Sciences, University of California, Davis, CA 95616 USA; 2 USDA Forest Service, Northern Research Station, Forestry Sciences Laboratory, Delaware, OH 43015 USA; 3 Department of Horticulture and Landscape Architecture, Purdue University, West Lafayette, Indiana, 47907 USA; 4 Present address: Division of Natural Sciences and Mathematics, Lewis-Clark State College, 500 8th Avenue, Lewiston, ID 83501 USA; 5 Schatz Center for Tree Molecular Genetics, Department of Ecosystem Science and Management, Pennsylvania State University, University Park, PA 16802 USA

**Keywords:** Beech bark disease, Association mapping, Resistance genes, American beech

## Abstract

**Background:**

The American Beech tree (*Fagus grandifolia* Ehrh.), native to eastern North America, is ecologically important and provides high quality wood products. This species is susceptible to beech bark disease (BBD) and is facing high rates of mortality in North America. The disease occurs from an interaction between the woolly beech scale insect (*Cryptococcus fagisuga*), one of two species of the fungus Neonectria (*N. faginata* or *N. ditissima*), and American Beech trees.

**Methods:**

In this case-control genome-wide association study (GWAS), we tested 16 K high quality SNPs using the Affymetrix Axiom 1.5 K – 50 K assay to genotype an association population of 514 individuals. We also conducted linkage analysis in a full-sib family of 115 individuals. Fisher’s exact test and logistic regression tests were performed to test associations between SNPs and phenotypes.

**Results:**

Association tests revealed four highly significant SNPs on chromosome (Chr) 5 for a single gene (*Mt*), which encodes a mRNA for metallothionein-like protein (metal ion binding) in *Fagus sylvatica*. Metallothioneins represent Cys-rich metal chelators able to coordinate metal atoms and may play an important role in the resistance mechanisms against beech scale insect.

**Conclusion:**

The GWAS study has identified a single locus of major effect contributing to beech bark disease resistance. Knowledge of this genetic locus contributing to resistance might be used in applied breeding, conservation and restoration programs.

**Electronic supplementary material:**

The online version of this article (doi:10.1186/s12864-017-3931-z) contains supplementary material, which is available to authorized users.

## Background

American beech (*Fagus grandifolia* Ehrl.) is native to the eastern North American deciduous forests and is the only species of this genus in North America [1]. The slow-growing, deciduous tree usually reaches about 37 m height (120 ft) and may attain ages of 300 to 400 years [2]. The native range of American beech is within an area from Nova Scotia in southeastern Canada, west to Wisconsin and south to eastern Texas and northern Florida in the United States. Beech wood is easily workable, excellent for turning and steam bending and used for flooring, furniture, veneer and containers [2].

Genetic research has been centered mostly on three genera (*Fagus*, *Castanea* and *Quercus*) of the family Fagaceae. The American beech (*Fagus grandifolia* Ehrh.) genome is estimated at 610 Mbp [3] and has yet to be sequenced. The number of chromosomes is generally stable within the Fagaceae family (2n = 24), with occasional changes (2n = 24 + 1, 2, 3) resulting from irregular segregation at mitosis [4]. Both *Fagus grandifolia* and *F. sylvatica* have the most rudimentary genomes within the family, making their genomes attractive for comparative genomics studies [3].

The thinness of beech bark makes it vulnerable to a range of scale insects. Beech bark disease (BBD) is a scale-fungal complex disease, initiated when a specific scale insect, *Cryptococcus fagisuga* Lind*.,* attacks the bark of beech trees and renders it susceptible to bark canker fungi of the genus *Neonectria* [5]. *Neonectria* (*Neonectria faginata* or *Neonectria ditissima*) is the most common genus of ascomycete fungi associated with beech bark disease [6–8]. The disease develops through feeding activity of the beech scale insect that creates points of entryway for the fungal pathogen. Once established on the tree, the fungus is no longer influenced by fluctuations in beech scale density caused by environmental factors or habitat quality [9]. The cankering response of beech to fungal infection produces bark microstructure fissures and when it progresses the cankers may coalesce girdling or partially girdling the tree leading to wilting foliage and even mortality of trees. Individuals that survive infection are more susceptible to abiotic and biotic stress [10].

Beech bark disease first occurred when the scale insect was accidently introduced on plant material imported to Nova Scotia from Europe around 1890 [11]. It is found throughout northeastern U.S. states and southeastern Canadian provenances and is still expanding. The disease distribution is generally attributed to the initial phase of the insect life cycle (“crawlers”) just after hatching, which is the only mobile phase of the life cycle. The “crawlers” can move to other areas on the same tree and can be dispersed by wind, birds, animals or humans. The mortality rate in beech has been significant in areas throughout the eastern United States. The loss of beech trees in some areas, where other hardwood species are rare, is causing even greater impact on wildlife, especially for black bear (*Ursus americanus*) [6, 12]. Pesticide control proved to not be fully effective in reducing the number of scale insects due to their protective waxy covering [13]. Neither pesticide application nor removal of infested trees is practical in large natural areas, because of labor, financial and environmental constraints [14]. Attempts to control *C. faggisuga* using a bio-agent such as a predatory mite [*Allothrombium mitchelli* Davis (Acari: Trombidiidae)] is currently under investigation [15].

Several field trials showed some beech trees remain after infestations that appear to be naturally resistant to BBD, usually clustered in small groups [16, 17]. About 1% of American beech trees remain disease free in forests long-affected by BBD [17]. There have been a small number of studies to understand the genetics of resistance of American beech to BBD [17–20]. Several studies have been conducted using molecular markers such as isozymes, RAPD (random amplified polymorphic DNA), AFLP (amplified fragment length polymorphism) and SSRs (simple sequence repeat) to differentiate between resistant and susceptible individuals and identify markers correlated with resistance, evaluate spatial and population genetic structure, and perform parentage analysis [19, 21–24]. Although genetic marker studies enhanced the efforts to clarify modes of inheritance, no markers have been discovered that correlate with resistance. To estimate heritability, resistance to the beech scale insect, the artificial infestation technique developed by Houston [16], was used to test parent trees and their full and half-sibling progeny for resistance [24]. Individuals were classified as susceptible if five or more scale insects were present on the bark surface 1 year after scale insect eggs were affixed to the test tree. Low levels of resistance were found in families with only one resistant parent and a higher proportion of resistant progeny were only observed in families where both parent trees were resistant, confirming that resistance to beech scale insect is a heritable trait [19, 24]. Current screening for resistance aims to increase the proportion of resistant trees and remove the susceptible trees for breeding purposes. These results formed the basis of a regional breeding program for BBD-resistant American beech [5, 25]. Identification of genetic markers associated with the resistance phenotype could potentially accelerate breeding efforts and reduce costs through the implementation of indirect selection methods, reducing the need for the costly, time consuming and labor intensive methods currently used to test trees for resistance to the scale insect.

Association mapping (AM) is an alternative approach that may, in theory, overcome limitations of pedigree-based quantitative trait loci (QTL) mapping [26]. It has been used in model species with available genomic resources, however with recently available genome sequences for forest species, this approach has the potential to increase the chance of understanding the genetic architecture of complex traits. The candidate-gene-based approach has been used for forest species where genome sequences were not yet developed [27]. In our study, we conducted a genome wide association study (GWAS) to discover loci associated with BBD resistance. A case-control design was used, which compared marker frequencies between a group of affected individuals (cases) and a group of unaffected individuals (controls). In this approach, we aimed to examine genetic architecture of disease resistance in American beech and identify candidate genes associated to BBD. Once identified, a marker breeding based approach could be used in breeding programs for BBD resistance.

## Methods

### Transcriptome

#### Plant materials

Trees sampled for RNA preparation were all part of the U.S. Forest Service Northern Research Station’s American beech breeding program in Delaware, OH. Tissues were sampled in the summer of 2009 from selected trees growing in naturally forested areas, grafted ramets of parent trees, and seedling progeny. The selected trees, or grafted ramets of the selected trees, were previously tested for beech scale resistance using an artificial infestation procedure [25]. To maximize genetic diversity, five resistant and five susceptible trees originating from a diverse geographic range, that included New Brunswick (Canada), Maine (USA), the lower peninsula of Michigan (USA), the upper peninsula of Michigan (USA), Pennsylvania (USA), and both northern and southern Ohio (USA) were selected for RNA sequencing and SNP discovery. Outer bark tissues including periderm, vascular cambium, and phloem, were harvested using grafting knives sterilized with liquid ethanol and were immediately frozen in a dry ice ethanol bath before transfer to a − 80 °C freezer until shipping overnight to the Schatz Center at Pennsylvania State University in a dry nitrogen shipper to prevent thawing.

#### RNA preparation

RNA samples were sequenced using Roche 454 sequencing technology. The main aim was to establish a database for network analysis to determine tissue specific expression patterns. Individual total RNA samples were prepared from bark tissues using the method first described by [28] and modified by [29]. At least five grams of frozen bark tissues were weighed, ground to a fine powder under liquid nitrogen, and dispersed in CTAB buffer. Following two chloroform extractions, RNA was precipitated with LiCl_2_, extracted again with chloroform and precipitated with ethanol. The resulting RNA pellet was re-suspended in 40–100 μl of DEPC-treated water, and the quality was assessed with an Agilent Technologies 2100 Bioanalyzer (Agilent Technologies). Poly(A) RNA was purified from total RNA using the Ovation RNA-Seq System kit (NuGen) following supplier’s instructions. Reverse-transcription was performed using the Just cDNA kit (Stratagene) and random hexamer primers.

#### Library construction and 454 Roche sequencing

Individual sequencing libraries for each cDNA preparation were constructed and sequenced using a 454 sequencer as previously described [29]. The cDNA preparations were sheared to approximately 500 bp fragment lengths. Adaptor sequences containing unique barcodes for each library were ligated to the fragmented cDNAs and immobilized on beads. The libraries for the five disease-resistant trees were pooled and the libraries for the five disease-susceptible trees were combined for separate multiplex sequencing, each on a different half of the same plate. Multiplex sequencing of the library pools was performed on an FLX model 454 DNA sequencer (Roche Diagnostics) at Penn State University. The DNA sequence files for each of the ten cDNA libraries were selected and compiled from the batch sequencing raw data files using a Newbler 454 software utility, based on the unique barcodes assigned to each of the libraries. Sequences generated in this study were submitted to the Short Read Archive at the National Center for Biotechnology Information, accessions numbers SRX1781388 to SRX1781397, for NCBI BioProject Accession PRJNA321730 (NCBI: http://www.ncbi.nlm.nih.gov).

#### Transcriptome assembly

The SeqMan NGEN (DNASTAR) program for next generation transcriptome sequence data assembly was used to assemble contigs from the pooled 454 sequence data files for all five BBD-resistant tree libraries. Similarly, the pooled dataset consisting of 454 sequence data files for the five BBD-susceptible tree libraries was also assembled using the NGEN program. Finally, to obtain a reference transcriptome for *F. grandifolia*, all of the 454 sequencing data files for the ten libraries were pooled and assembled into a combined set of transcript contigs.

#### Filtering of transcriptome assembly and SNP calling

Mapping of the reads from each library onto the assembled transcriptomes revealed a high level of several sequences of ribosomal origins. These transcripts accounted for 34% (180,292 out of 533,261), 27% (139,718 out of 521,505) and 18% (186,745 out of 1,026,995) of the resistant, susceptible and combined assemblies, respectively. To determine the amount of structural non-coding RNA (ncRNA) sequences present in the libraries, the RFAM structural RNA database [30] was downloaded to serve as a local BLAST database. All of the sequence reads and contig sequences in the three assembled transcriptomes were aligned to the RFAM database, using a conservative BLAST *e*-value threshold of e-70, the ribosomal content was about 30% of the sequenced reads. ncRNAs accounted for 729, 1506 and 2110 contigs for the resistant, susceptible and combined assemblies, respectively. These contigs were removed from the assemblies, prior to SNP discovery.

Due to the large amount of ncRNA reads, another assembly was conducted using the Newbler program (Roche) which incorporated filtering for structural RNAs, low quality reads, and abbreviated reads. The BLAST cut-off *e*-value used in the filtering ncRNAs was set at e-50, resulting in 349,613 out of 1,406,316 reads in total to be removed prior to assembly. Again, assemblies were conducted for the 5 BBD-resistant libraries, the 5 BBD-susceptible libraries, and all 10 libraries combined.

Detection of putative polymorphic sites was performed by mapping reads filtered for structural RNAs to the beech reference transcript contigs, using the Newbler’s *gsMapper* program. Both DNASTAR and Newbler all-library combined contig sets were used, separately, as reference transcriptomes in the SNP discovery. A minimum depth of coverage of 15 reads from each library was required to call putative SNPs. High confidence SNP sites were identified as those with a minimum of 100 bases flanking the SNP site and where the reference nucleotide at the SNP site was non-ambiguous. The stress-response genes and EST-based DNA markers served as a resource (*Fagus grandifolia* Transcriptome, Hardwood Genomics Project, www.hardwoodgenomics.org) for the construction of linkage maps and a framework for a GWAS study.

### Population sampling and phenotypic classification

**Fig. 1:**
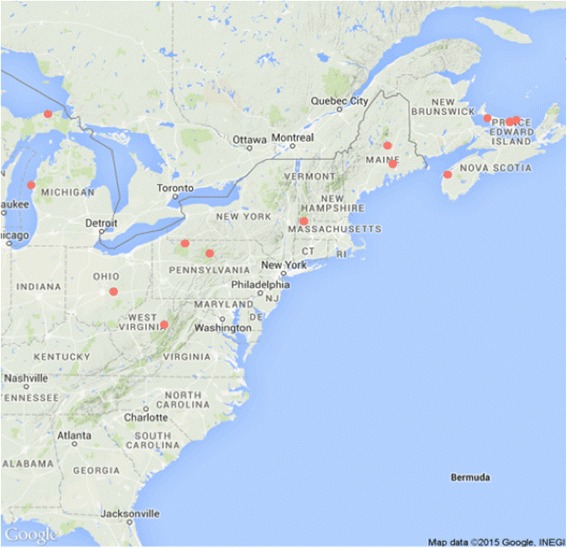
Sampling locations of mapping population. Highlighted by *red full circles* are sampling locations of American beech full-sib individuals used for association mapping study across stands in six U.S. states and two Canadian provinces. The map graphic was reproduced in the package ‘ggmap’ (Spatial visualization in ggplot2) v.2.6.1 (Kahle and Wickham) [38] in software R with Google Maps and Stamen Maps. For Fig. 1 a copyright permission was not required

Resistant trees (*R* = 254) and susceptible trees (S = 260) were located and positions mapped in six US states and nine stands. Trees in Penobscot county (Maine), Berkshire county (MA), Randolph county (WV) and Pascataquis county (ME) and two Canadian provenances: Prince Edward Isl. Canada, Kings county, and Sissiboo Falls, Digby county (NS) were located and mapped as reported in previous studies [18, 22]. Additional trees were located and mapped in Ludington State Park, Mason county (MI), Luce county (MI), Mckean county (PA), Clearfield county (PA), Clinton county (PA) and Licking county (OH) (Fig. 1).

Phenotypes were determined for 514 individuals through field assessment of all trees at the time of tissue collection. Trees exempt of beech scale insects and any apparent signs of fungal infection were classified as resistant. A subset of trees was artificially inoculated with scale eggs to confirm resistance either in the field or on grafted ramets of the original trees [16, 17, 25]. A complete description of sampled trees and their phenotypes is included in Additional file 1.

### Tests of association

#### SNP genotyping

Extraction of DNA from leaf and dormant bud tissues was carried out as described in [19]. Dormant buds were collected as described in [18] and stored at −70 °C until used for DNA extractions. For the 514 samples, DNA quality and quantity was tested before submitting for genotyping. Approximately 20 K SNPs, originating from EST transcriptome assemblies, were submitted for design scores and 16 K of these were selected for inclusion on an Affymetrix Axiom™ Genome-Wide 1.5 K - 50 K array (Santa Clara, CA). Genotypes were successfully determined for 506 of the 514 samples and were used in downstream analysis with an initial call rate of 97% or greater.

#### SNP linkage map

Controlled cross-pollinations were carried out between two confirmed beech scale resistant American beech trees 1505 (R) and 1504 (R) in Ludington State Park, MI (USA) as previously described to create the mapping population [31]. Additional cross-pollinations were carried out on grafted containerized ramets of the parents in 2010 to supplement the original family (*N* = 46), bringing the total number of progeny to 117.

Linkage analysis was performed on 115 of the 117 progeny that were successfully genotyped at 5838 SNPs sites. A genetic map was constructed to order SNP markers used in association testing and served as graphical displays of the genome wide significant associated SNPs. The map construction was performed using the software Join-Map 2.0 [32]. All SNPs that successfully “passed” a 1:2:1 or 1:1 segregation ratio test in the offspring, were used in the linkage analysis, assigned to linkage groups and ordered to determine map genetic distances in cM (centimorgans).

#### Population structure analyses and genome-wide identity-by-state test (IBS test)

Discriminant Analysis of Principal Components (DAPC) was used to cluster individuals based on genotypes. This well-known method aims to maximize group differences while minimizing within cluster variances [33]. DAPC was applied on a matrix composed of 506 individuals and 5838 SNPs using an implementation available in the R package *adegenet* 2.0.0. To identify clusters, the principal component analysis (PCA) of the matrix data was first computed followed by Discriminant Analysis on the number of retained principal components as provided by DAPC method.

A test for population stratification was also performed with *qqman* package [34] in R software v.3.2.0, a common tool to visualize GWAS results and estimate the rate of genomic inflation. We measured genomic inflation also defined as λ (lambda) to provide evidence of population stratification or cryptic relatedness.

To avoid statistical biases during population structure analysis and IBS score computations, SNPs with Pearson’s correlation coefficients (r^2^) higher than 0.8 were rejected. Due to the absence of a genetic scaffold, a chromosomal location-based SNP filtering, as implemented in common GWAS packages, such as PLINK v.1.9 [35], was replaced by iterative SNP filtering. Pairwise Pearson’s r^2^ across SNPs were computed and, at each step during the iteration, the SNP correlated (r^2^ > 0.8) with the most number of other SNPs was rejected. The iteration stopped when no remaining SNPs were correlated (all pairwise r^2^ < 0.8). This procedure left 3220 SNPs out of original 5838 SNPs. Filtered SNPs were then used to compute IBS scores, namely pairwise Pearson’s correlation coefficients (r^2^), between individuals. Here again iterative filtering was applied across trees to prune individuals with IBS > 0.1875. Out of 506 trees, 327 passed IBS-based filtering.

#### GWAS

A logistic regression model was used to perform association tests between SNPs and disease scores. Population stratification was controlled using the first 20 ancestry principal components (PC’s) as covariates in the logistic model. GWAS was performed using the PLINK 1.9 package ([35], https://www.cog-genomics.org/plink2, [36]) and 3220 SNPs on 327 independent individuals (172 cases and 155 controls, all pairwise IBS < 0.185). Prior to association testing, individuals with missing genotype rate > 10% were deleted, leaving effectively 172 cases (susceptible or diseased trees) and 155 controls (resistant or symptom-free trees). SNPs were then filtered for Hardy-Weinberg equilibrium (*p*-values >10^−5^), minor allele frequencies (MAF > 0.05) and missing genotypes across individuals (< 10%). In total, 3155 SNPs were included in the association test after filtering. The genotyping rate was equal to 0.99. For every SNP included in the case-control test, the exact *P* value [P] and the estimated odds ratio [OD] for the association between the minor allele [A1] and the disease phenotype were calculated. Resulting *p*-values underwent genomic inflation control.

#### Linkage disequilibrium

Haplotypes were identified using Haploview (v.4.2) [37], and with default parameters (exclusion of markers separated by >500 kb and individuals with >50% missing genotype). Pearson’s coefficient of determination (r^2^) was used to determine the pairwise correlation between genetic markers.

## Results

### Transcriptome results

#### RNA sequence data

The sequencing of 10 cDNA libraries yielded 1,406,316 reads covering 508,764,432 bases. The libraries from BBD-resistant trees yielded between 87,964 and 210,340 reads, while 70,218 to 205,945 reads were obtained for libraries from BBD-susceptible trees (Table 1). While the quality and quantity of the sequence data was acceptable (Table 1; Additional file 2), the mRNA poly-A selection step left a large proportion of structural non-coding RNAs in the samples, including ribosomal RNA. Moreover, up to a third of the transcriptome sequence reads were mapped to ncRNAs. Subsequently, the quality of the mRNA preparations was re-assessed with an Agilent Technologies 2100 Bioanalyzer (Agilent Technologies), revealing remnant rRNA peaks (Additional file 3). Because the ncRNA contamination was detected after the first transcript assemblies were conducted, a second assembly with additional filtering was conducted (see below).

**Table 1:** Result metrics for the sequencing of 10 beech cDNA libraries

Library Name (based on tree number)	Number of reads	Average read length	Total bases
BBD-Resistant trees			
Beech_1228R	210,340	375	78,912,594
Beech_2692R	131,306	354	46,467,779
Beech_1504R	123,194	370	45,498,709
Beech_1208R	87,964	358	31,467,605
Beech_2276R	147,781	374	55,192,118
Resistant tree Sub-Totals	700,585	366.2	257,538,805
BBD-Susceptible trees			
Beech_1973S	205,945	358	73,702,265
Beech_DN00726S	159,660	349	55,759,976
Beech_3128S	130,917	359	46,933,567
Beech_2143S	138,991	357	49,586,742
Beech_Holden	70,218	360	25,243,077
Susceptible tree Sub-Totals	705,731	356.6	251,225,627
Totals for all libraries	1,406,316	361	508,764,432

#### Assembly of transcript contigs

Assemblies of 454 sequence reads into contigs were conducted using both the SeqMan NGEN (DNASTAR) program and the 454 Newbler (Roche) assembler. Contigs were built using reads from, either five BB-resistant libraries, five BBD-susceptible libraries or combination of both libraries.

The NGEN assembly resulted in a total of 28,592, 27,544 and 44,065 contigs for pools of BBD-resistant, BBD-susceptible and the combination of both libraries, respectively. NGEN assemblies incorporated 76%, 73% and 73% of the sequence reads from the BBD-resistant, BBD-susceptible and the combined samples. NGEN contig lengths averaged approximately 360 bases, with a median varying from 475 to 542 bases. The longest transcripts obtained in the resistant, susceptible and combined assemblies were 11,704, 8168 and 10,800 bases, respectively. Table 2 summarizes the results of the three *F. grandifolia* transcriptome assemblies using the NGEN program.

**Table 2:** *F. grandifolia* transcriptome NGEN assembly statistics

	BBD-Resistance Libraries	BBD-Susceptible Libraries	“Combined” Reference transcriptome
Assembled Reads	533,261	521,505	1,026,995
Unassembled Reads	167,324	184,226	379,321
Total Number of Reads	700,585	705,731	1,406,316
Assembled Reads (%)	76.12	73.90	73.03
Assembled Contigs	28,592	27,544	44,065
Contigs >2 K	622	271	1115
Av. Length of Contigs	362	354	357

The Newbler filtered assembly resulted in 10,690 contigs for the BBD-resistant data, 7630 contigs for the BBD-susceptible data, and 16,285 contigs for all libraries combined. The Newbler assemblies incorporated 84%, 81% and 86% of the sequence reads from the BBD-resistant, BBD-susceptible and the combined data, respectively. The longest transcripts obtained by Newbler in the resistant, susceptible and combined assemblies were 4651, 4336 and 10,681 bases respectively. The average length of the Newbler contigs across the three assemblies was 679 bases. The detailed Newbler assembly statistics are shown in Table 3. The “large contigs” from the Newbler assembly are those with 500 bases or longer, which overall averaged 949 bases in length.

**Table 3:** *F. grandifolia* transcriptome sequence assembly summary obtained from Newbler

Assembled Contig Sequences	Beech “Resistant” Library	Beech “Non-Resistant” Library	Beech “Combined” Library
Aligned Bases (%)	157,365,926 (83.63%)	143,162,558 (80.91%)	314,816,063 (86.23%)
Aligned Reads (%)	438,153 (82.04%)	412,164 (78.90%)	893,216 (84.54%)
Number of Contigs, All	10,690	7630	16,285
Total Contig Bases	7,845,700	4,478,078	11,664,012
Average Contig Length	734	587	716
Number of Large Contigs	7147	4018	9943
Average Large Contig Size	961	875	1009
N50 Large Contig Size	1005	893	1081
Largest Contig Size	4651	4336	10,681

#### Single nucleotide polymorphism (SNP) discovery

SNP site discovery was performed using both the DNASTAR NGEN and Newbler combined assemblies as reference transcriptomes. The Newbler *gsMapper* SNP calling program generated two output files. One output file contained all possible SNPs. The other output file contained only the high confidence SNP calls, which was used as the starting point for selecting SNPs for the mapping study. A summary of the SNP discovery results is displayed in Table 4. As shown in the last two columns of Table 4, 15,542 and 12,119 candidate SNP sites were discovered using the Newbler and DNASTAR reference transcriptomes, respectively. For each candidate SNP with a minimum 100 bases of sequence flanking the SNP, 50 bases from each flanking side of the SNP site was extracted for DNA marker development. The values in parenthesis in the last two rows of Table 4 represent the number of SNP sites selected, based on sufficient flanking sequence. The sequences and statistics for the high quality and most informative SNPs used in the GWAS are presented in a table in Additional file 4.

**Table 4:** Summary of SNP discovery results for *F. grandifolia* using the reference transcriptomes generated by DNASTAR NGEN and Newbler

Statistics	DNASTAR Reference Transcriptome	Newbler Reference Transcriptome
Number of Contigs in Reference	43,212	14,977
Number of Bases in Reference	28,676,242	11,580,835
Number of Mapped Reads (%)	1,357,629 (96.55%)	938,418 (88.82%)
Number of Mapped Bases (%)	494,805,288 (97.45%)	312,081,222 (85.48%)
Fully Mapped Reads (%)	444,838 (31.64%)	396,609 (37.54%)
Partially Mapped Reads (%)	35,728 (2.54%)	135,858 (12.86%)
Non-Unique Mapped Reads (%)	871,685 (61.99%)	147,210 (13.93%)
Chimeric Reads (%)	5378 (0.38%)	258,741 (24.49%)
Unmapped Reads (%)	32,025 (2.28%)	101,623 (9.62%)
Reads Too Short (%)	16,470 (1.17%)	16,470 (1.56%)
High Confidence SNP Calls	2119 (12,069)	15,542 (14,574)
HC SNP Calls (Not Ambiguous)	10,971 (10,934)	15,541 (14,573)

### Test of association

#### SNP genotyping data results

In total, 514 DNA samples were submitted to genotyping using the Affymetrix Axiom™ Genome-Wide 1.5 K - 50 K array and after quality filtering, resulted in genotypes for 506 samples (*R* = 249 and S = 257). Of the initial 16,709 SNPs, 5838 Poly High Resolution SNPs passed Affymetrix filtering metrics and only these were included in downstream analysis. A set of 5838 SNPs was visualized for the cluster pattern in SNPolisher R Package v.1.5.1 (Affymetrix Inc.).

#### SNP linkage map construction

**Fig. 2:**
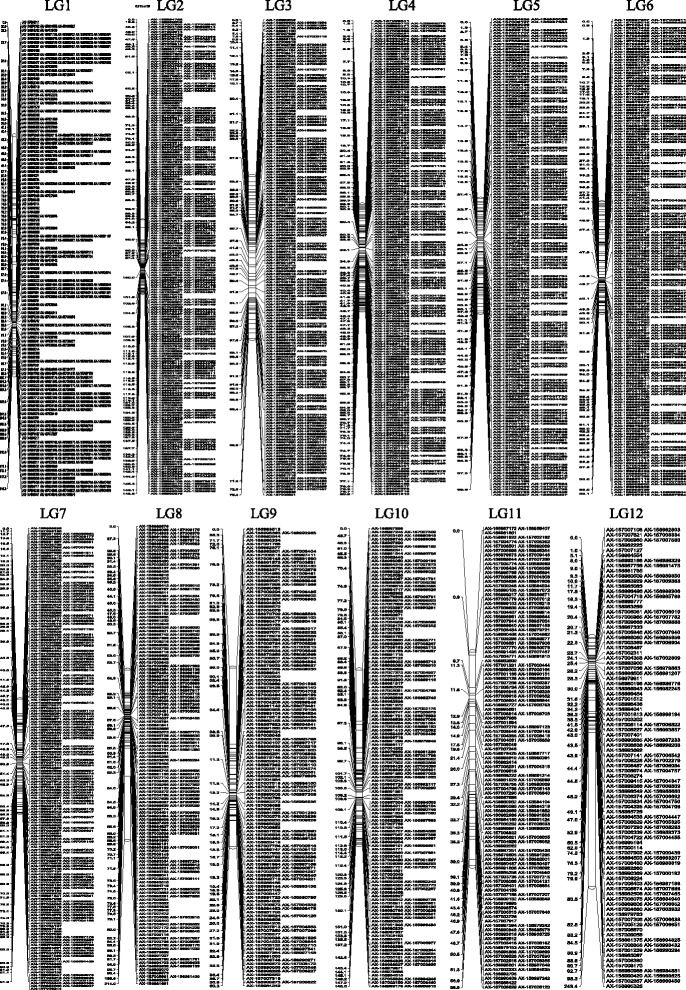
Genetic linkage map of *F. grandifolia.* Genetic linkage map of *F. grandifolia* constructed using 115 progeny individuals derived from the cross controlled experiment 1505 (R) × 1504 (R). Totally 3220 single nucleotide polymorphism markers are linked in twelve groups and presented on the right side of each linkage group. Map distances in centi-morgans are presented on the left side

We developed a single-nucleotide polymorphism (SNP) - based linkage genetic map for American beech. Single locus Mendelian segregation was first tested using *X* ^2^ goodness-of-fit to 1:2:1 and 1:1 ratio at 5% and 1% significance levels. Linkage analysis produced 12 linkage groups (Fig. 2) using JoinMap 2.0 [32]. Out of 3236 SNPs apparently segregating, 16 SNPs failed to be linked so the final number of linked SNPs was 3220 (Additional file 5).

### GWAS

#### Population structure analysis and IBS test results

To explore the population structure of our sample population, Discriminant Analysis of Principal Components (DAPC) was applied to 506 individuals and 5838 SNPs. DAPC revealed three genetic clusters (Additional file 6A) using 40 principal components (PCs), maximum numbers of clusters and discovery clusters limited to 40 and 7, respectively and 6 discriminants. In addition, we employed a genomic control to assure for population structure by estimating an inflation factor λ (genomic control measures). Significant inflation was detected based on a QQ-plot of association *p*-values, which displayed systematic deviation from the expectation (Additional file 6B).

The pairwise clustering based on identity-by-state (IBS), revealed high correlation among individuals in the sample population. The IBS test allowed the removal of individuals with the highest number of correlated “partners”, indicating high likelihood of relatedness. Our method of choice was to test for population outliers by performing IBS-based nearest neighbor analysis. In total, 179 individuals from the five different stands were identified as possible very close relatives and were removed from the further downstream analysis (Table 5).

**Table 5:** Duplicated individuals revealed by IBS test for the threshold (IBS > 0.1875)

Stand	Number of excluded individuals	Disease status	
Ludington State, MI	1	R	NA
Berkshire county, MA	18	9R	9S
Penobscot county, Maine	55	33R	22S
Randolph county, WV	38	26R	12S
Sissiboo Falls, Digby county, NS-Canada	65	27R	38S
Not classified	2	NA	2S
Total	179		

#### Logistic regression test

**Fig. 3:**
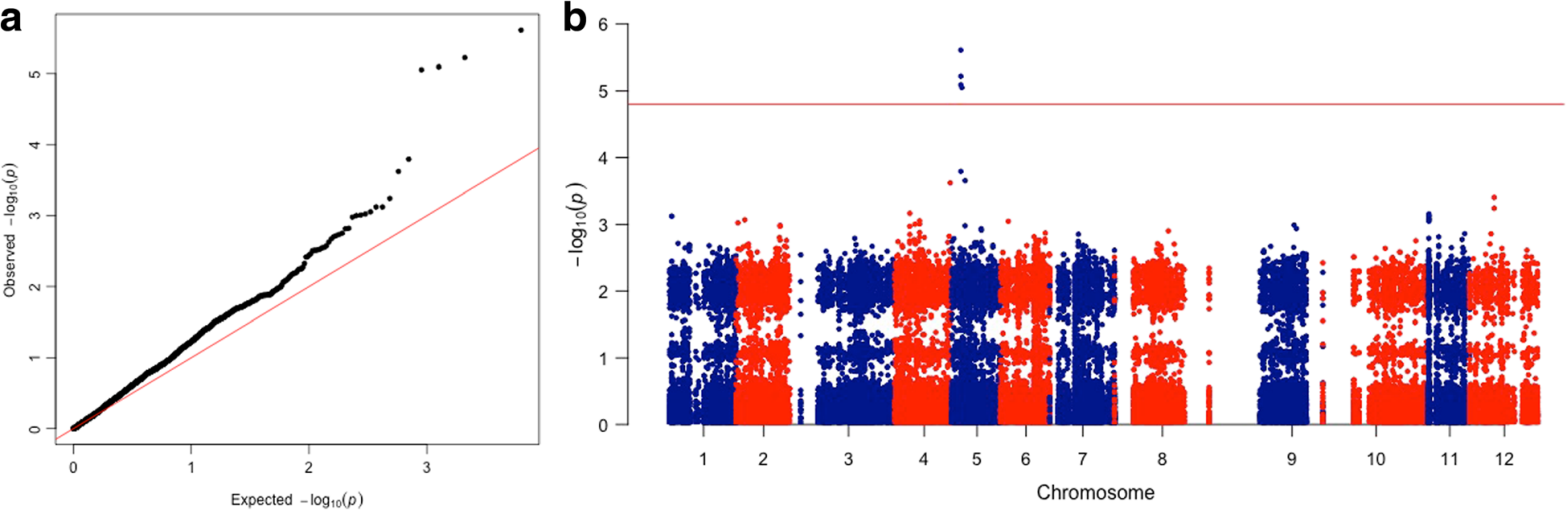
**a** Quantile-quantile (QQ) plot of GWA *p*-values. QQ-plot shows only minor deviations from the null distribution, expected for the top associated SNPs. **b** Manhattan plot from the GWAS analysis of Beech Bark Disease in 327 individuals. Beech bark disease is associated with a locus on chromosome 5. The x-axis represents chromosomal locations and the y-axis, −log10 *p-*values from genotypic associations. Four markers on chromosome 5 reached genome-wide significance (*p*-values >1.585 × 10^−5^)

The initial logistic regression test was performed with 3220 SNPs, however after filtering SNPs to compute PCA and IBS score, an independent set of 2116 SNPs remained. No Genomic inflation from GWAS *p*-values expected by random chances was detected, except for the top associated SNPs (lambda = 1.13, Fig. 3a). A Fisher’s exact test revealed four markers on chromosome 5 (Fig. 3c), whose *P* values were above the significant genome-wide threshold of (*P* value >1.585 × 10^−5^) (Fig. 3b; Additional file 7). For the association test, the significance threshold for all 3155 SNPs was Bonferroni’s (α^*^ = α/n) based significant threshold to adjust for multiple testing, where α represents Bonferroni’s coefficient 0.05 and *n* represents the number of SNPs after filtering for quality parameters (0.05 / 3155 = 1.58 × 10^−5^).

#### Identification and mapping of the disease resistance candidate gene

As shown in the Manhattan plot (Fig. 3b and Additional file 7), four SNPs were observed to surpass the genome-wide significance threshold of 1.585 × 10^−5^, which is strong evidence of association. All four SNPs are located on chromosome (Chr) 5 (Additional file 8). The strongest evidence of association is linked to AX-156994126 (*P* = 5.99E-6, odds ratio (OR) = 0.2573), AX-156988334 (*P* = 8.852E-6, odds ratio (OR) = 0.2758) and AX-157000652 (*P* = 8.074E-6, odds ratio (OR) = 0.2773) (Table 6). On chromosome (Chr) 5, SNPs were positioned at 12.344 cM (centimorgans) for AX-156994126, AX-156989406 and for AX-157000652 and at 13.811 cM for AX-156988334 (Additional file 8A).

**Table 6:** Top SNPs associated with Beech Bark disease

Gene	Chr	Position (cM)	Affymetrix ID	Original SNPs ID	Logistic regression association (*P* value)	Annotation
Mt	5	12.344	AX-156989406	contig03321_576	2.46E-6	*Fagus sylvatica* (European beech) mRNA for methallothionein-like protein, Metal ion binding
Mt	5	12.344	AX-157000652	contig03321_166	8.07E-6	
Mt	5	13.811	AX-156988334	contig03321_330	8.85E-6	
Mt	5	12.344	AX-156994126	contig03321_441	5.99E-6	

The flanking sequences for these four SNPs were used in Blast analysis. The best BLAST (BLASTn) analyses were performed against the NCBI database (National Center for Biotechnology Information) for non-redundant protein database. The best hit resulted in the identification of the single gene (*Mt*) from a single contig (contig 03321), within which fell all four identified SNPs (see Additional file 8B). The gene (*Mt*) encodes an mRNA for metallothionein-like protein (metal ion binding) (Table 6).

Support for the result from GWAS was obtained by BLASTx alignment of the RNA sequence reads for each of the 10cDNA libraries individually to contig 03321, containing the full length transcript of the candidate *Mt.* gene. With the exception of the individuals (1504R and DN00726S), the constitutive expression of the candidate *Mt.* gene was higher in the BBD resistant individuals than in the BBD susceptible individuals (Additional file 9). On average, 1602 reads mapped per BBD resistant library, and 414 reads per susceptible library, after normalization in TPM (Transcripts Per Kilobase Million). This does not imply the expression of the candidate *Mt.* gene alone is sufficient for BBD resistance, nor that it is the only gene differentially expressed upon attack by the insect vector.

#### Linkage disequilibrium

To measure the degree to which alleles at two loci are associated, a complete set of 3220 SNPs were included to determine whether two loci are in linkage equilibrium or disequilibrium. LD plot showed SNPs in strong linkage disequilibrium (D’ = 1) (Additional file 10).

## Discussion

### Trait architecture

Genome Wide Association Analysis has identified a single locus contributing to resistance to beech bark disease (BBD). There were four SNPs in chromosome (Chr) 5 significantly associated with the scale resistance trait analyzed. A candidate gene (*Mt*) encoding for a metallothionein-like protein was found to be physically linked to these genetic markers and may play an important role in the resistance mechanisms against *Nectria* sp. - beech scale insect. This is consistent with genetic studies of several different small full-sibling families that suggest involvement of a few as two genes [5, 24, 38]. For validation of single locus trait discovery, BLASTn search of the contig EST sequences was performed against the complete NCBI database for those SNPs (see Additional file 8B). A proven functional annotation for these SNPs is essential prior to use in breeding, which will be possible when a reference genome sequence for American Beech is available.

Disease resistance in plants can involve any number of genes, from a single major gene to many loci determining resistance. Single-gene resistance mechanisms with large effects are more common in agricultural crops but only a few have been described in forest species, which reflects the greater genetic diversity of the host and pathogen populations in forest pathosystems [39]. In forest species, resistance is typically polygenic and durable, with few examples of simply inherited disease resistance. This is likely due to the limited potential for Mendelian analysis in forest trees and complex life cycles of many forest pathogens [40]. However, disease resistance is not exclusively polygenic in forest pathosystems. Examples of single qualitative resistance, include loblolly pine (*Pinus taeda*) resistance to the fusiform rust disease [41, 42], resistance to white pine blister rust in several species of pine [43, 44] and evidence for major gene resistance to weevil in Sitka spruce [45].

In this study, we used Affymetrix Axiom™ Genome-Wide 1.5 K – 50 K array (Santa Clara, CA) to genotype 327 individuals used for association mapping. Although SNP discovery was performed specifically for BBD, a very small proportion of the SNPs deemed informative in downstream analysis. The conversion rate, provided by Affymetrix genotypng facility, corresponded to 34.04% and was quite high compared to *Pinus taeda* (Loblolly pine) at only 5–10%. Overall, the number of informative SNPs was sufficiently high to provide us with association power on the genome scale for the disease resistance.

The knowledge of genetic architecture is important for breeding resistant varieties to develop resistant planting stock for restoration of impacted habitats. Molecular markers have also contributed to improved breeding strategies for monogenic resistance genes when combining them in a “gene pyramiding” strategy for a more durable resistance [46] and can also be used to develop cost-effective indirect selection techniques.

### Candidate gene role

Plant metallothioneins are proteins thought to sequester excess amounts of certain metal ions [47]. These low molecular weight proteins (4–8 kDa) were discovered in mature wheat embryos about 30 years ago [48]. Metallothioneins represent Cys-rich metal chelators able to coordinate metals atoms (e.g. Zn, Cd and Cu ions) and found to play a role in cellular processes such as regulation of cell growth, proliferation and DNA damage repair. But how metallothioneins fulfill these cellular roles, is yet to be discovered [49, 50]. Expression of plant metallothionein genes has been observed in a variety of senescing tissues, such as leaves and stems, ripened fruits and wounded tissues [49]. Recent reports show that MTs (metallothionein’s) are also involved in the scavenging of reactive oxygen species (ROS) [51].

Metallothionein-like protein class II (*Fagus sylvatica* type) was described in Norway spruce (*Picea abies)*, whose expression pattern was analyzed via ESTs from cDNA libraries [52]. Type 4 metallothionein-like protein genes are expressed in inner bark tissue of Japanese cedar (*Cryptomeria japonica*) [53]. ESTs encoding metallothionein-like proteins were the most frequently found hits in both early and late flushing libraries. Metallothionein-like protein activity is probably initiated by some cellular events during late flushing [52].

Changes in expression of metallothioneins and metallothionein-like proteins have been previously reported in response to biotic stresses in plants, including insect herbivory and fungal infections (reviewed in [53]). There is not consensus of the role of metallothionein-like proteins in biotic stress response, but a role in oxidative stress have been proposed [53].

### Implementation of the findings in the future breeding program in beech

A number of insects and diseases cause significant loss to forest productivity. Most of the current operational strategies for insect and disease control rely on classical breeding methods to develop populations enriched for resistance [54]. With emergence of genomics-based approaches, such as genome-wide association studies (GWAS) and genomic selection (GS), a broader range of applications is now available for plant breeding and genetic research [55, 56]. In *Fagus*, mapping populations have been developed to discover QTLs for traits correlated to BBD. The tree improvement program included crosses to study inheritance of resistance to *Cryptococcus fagisuga* (see [19, 57]). However, association mapping like GWAS for QTLs underlying disease resistance to the BBD, has not been previously reported. In the present study, we used a GWAS mapping approach and a SNP linkage map to identify candidate resistance genes. To confirm the SNPs identified are truly associated with the scale-resistant trait, replication of this GWAS study is necessary, using independent case-control data from the initial population of unrelated individuals (see [58, 59]).

Deployment of resistant planting stock can help to reduce disease incidence throughout natural stands of American beech. Markers found in this study that exhibit a significant association with the resistant phenotype, can be further refined to develop efficient and cost effective indirect selection techniques such as MAS (marker assisted selection) and genomic selection (GS) or combination of both (see [60]).

## Conclusion

To our knowledge, this is the first study designed to determine the genetic factors of disease resistance to beech bark disease (BBD) with genome scan analysis in American beech tree. The results presented identified four highly significant markers associated with a single locus located on chromosome (Chr) 5. All four loci were localized to the same contig within a single gene (*Mt*), that encodes for *Fagus sylvatica* mRNA for metallothionein-like protein (metal ion binding). Once a reference genome sequence is available, it will be possible to gain more insight into functional annotation of the four SNPs and determine the exact number of genes associated to BBD.

## Additional files

Additional file 1: The list of sampled trees and their phenotypes. (XLSX 146 kb)

Additional file 2: RNA‐seq data quality assessment. (PDF 853 kb)

Additional file 3: Examples of RNA vs. mRNA Agilent Bioanalyzer quality profiles. (PDF 288 kb)

Additional file 4: The sequences and statistics for the high quality and most informative SNPs used in the GWAS. (XLSX 1080 kb)

Additional file 5: The list of mapped SNPs in 12 linkage groups (LGs). (XLSX 91 kb)

Additional file 6: (A) DAPC analysis revealed three main genetic clusters where the individuals shown as dots and the groups as inertia ellipses. Eigenvalues of the analyses are displayed inset. (B) Quantile-quantile (QQ) plot of GWA *p*-values where on x-axis, are expected –log10 *P* values and on y-axis observed –log10 *P* values. The plot is showing large deviation from the null distribution where the inflation factor was higher than the threshold of 1, indicating a high genomic inflation in Beech association data and an existence of the population stratification. (PDF 90 kb)

Additional file 7: The statistics for Fisher exact test and Logistic regression test. (XLSX 4534 kb)

Additional file 8: (A) Four highlighted markers with significance level higher than genome-wide threshold (*P* value >1.585 × 10^–﻿﻿5^﻿) located on the chromosome (Chr) 5. (B) Alignment of four nucleotide sequences to reference sequence *Fagus sylvatica* mRNA (Sequence ID: AJ130886.1). The FASTA sequence order corresponds as AX-156994126 (SEQ_1), AX-156989406 (SEQ_2), AX-156988334 (SEQ_3) and AX-157000652 (SEQ_4). Highlighted red nucleotides refer to polymorphism to reference sequence and green nucleotides present diagnostics SNPs, respectively. (DOCX 4155 kb)

Additional file 9: RNA sequence reads from each cDNA library mapped to the full-length copy of the candidate gene transcript sequence from contig 03321, representing the expression of the candidate *Mt* gene after the challenge by the insect vector. (DOCX 161 kb)

Additional file 10: Linkage disequilibrium (LD) structure across four SNPs associated to BBD. (Red) strong LD between markers; (white) no LD. The block-like pattern represents the regions of high LD. Pairwise LD among four SNPs listed as squared allelic correlation (r2) [61] and Lewontin’s D’ [62]. (DOCX 162 kb)

## Abbreviations

AFLP: Amplified fragment length polymorphism
AM: Association mapping
ARE: Antioxidant
BBD: Beech bark disease
BLAST: Basic alignment search tool
BLASTn: Nucleotide basic alignment search tool
cDNA: Complimentary DNA
Chr: Chromosome
cM: Centimorgans
CTAB: Cetyltrimethyl ammonium bromide
DAPC: Discriminant analysis of principal components
DEPC: Diethyl dicarbonate or diethyl purocarbonate
ERE: Ethylene
EST: Expressed sequence tag
GARE: Gibberellic acid
GS: Genomic selection
GWAS: Genome-wide association study
IBS: Identity-by-state
LD: Linkage disequilibrium
LiCl_2_: Lithium chloride
MAF: Minor allele frequencies
MAS: Marker assisted selection
MeJARE: Methyl jasmonate
*Mt.*: Metallothionein-like protein (metal ion binding)
NCBI: National Center for Biotechnology Information
ncRNA: Non-coding RNA
OD: Odds ratio
PCA: Principal component analysis
PCs: Principal components
Poly(A)RNA: Polyadenylation RNA
Q-Q plot: Quantile-quantile plot
QTL: Quantitative trait loci
RAPD: Random amplified polymorphic DNA
rRNA: Ribosomal nucleic acid
SARE: Salicylic acid
SSRs: Simple sequence repeat
TPM: Transcripts Per Kilobase Million
λ: Lambda
