# Supplementary material for: Genome-wide association study identifies a major gene for beech bark disease resistance in American beech (Fagus grandifolia Ehrh.)
Source: BMC Genomics. 2017 Jul 20;18:547. doi: 10.1186/s12864-017-3931-z (PMC5520234; doi:10.1186/s12864-017-3931-z)

RNA-seq data quality assessment

1208 Resistant

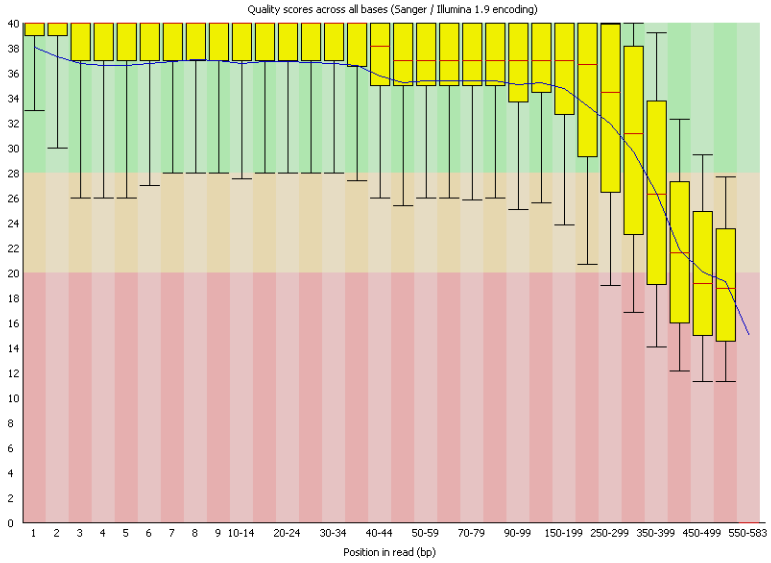

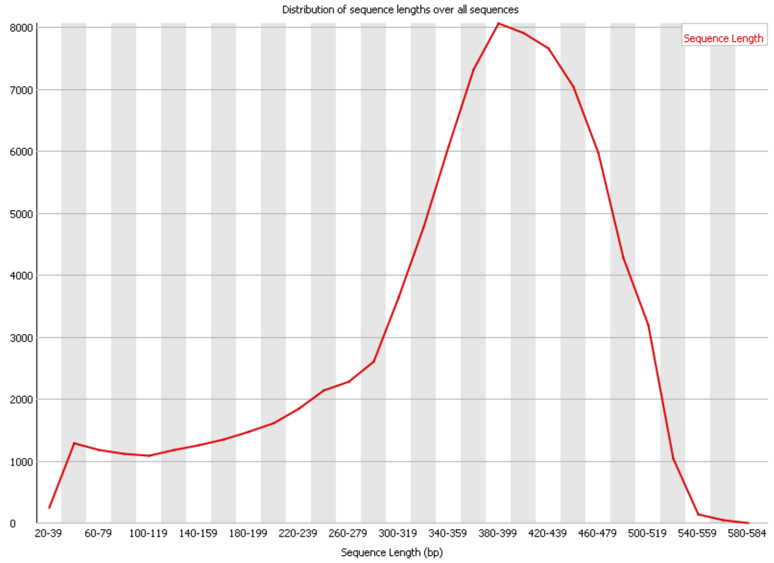

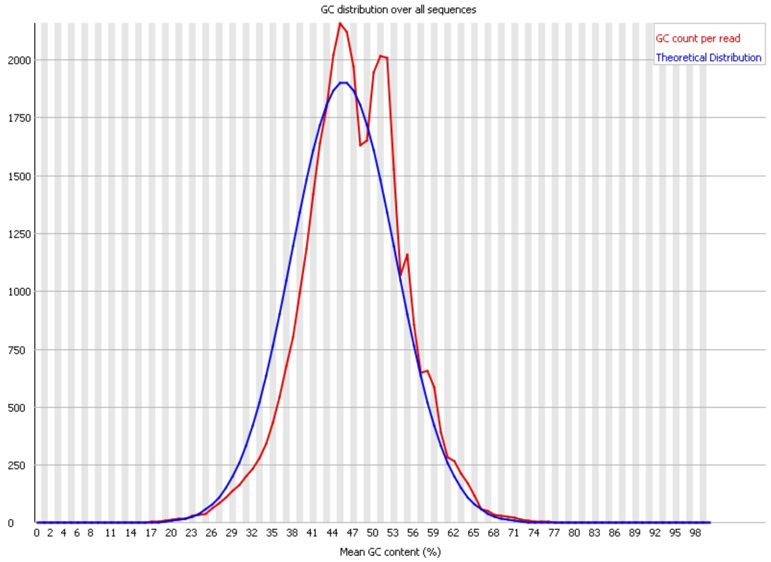

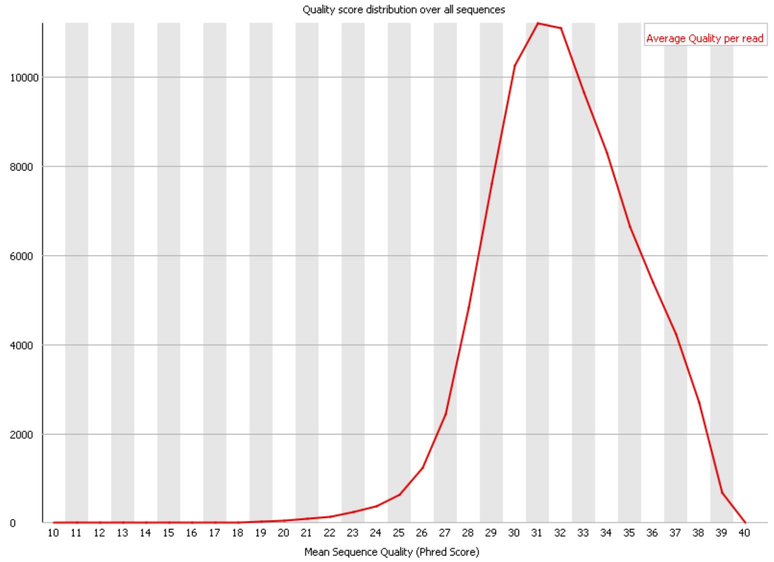

RNA-seq data quality assessment

1504 Resistant

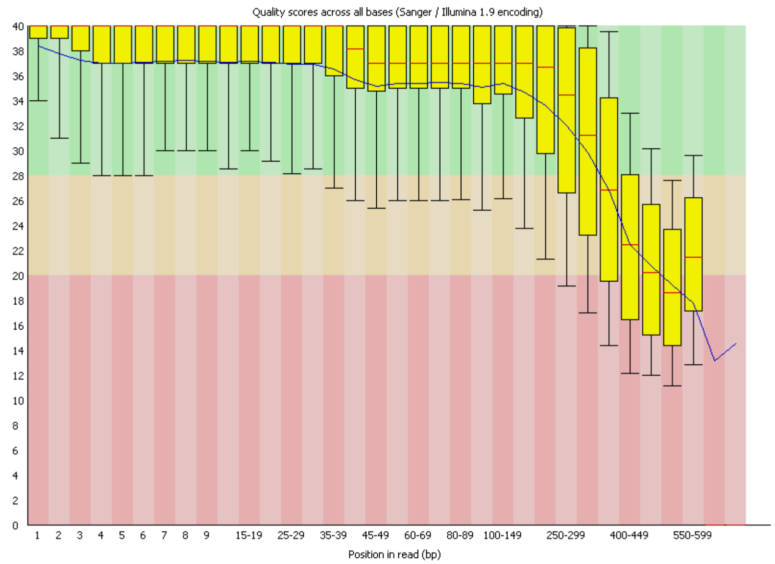

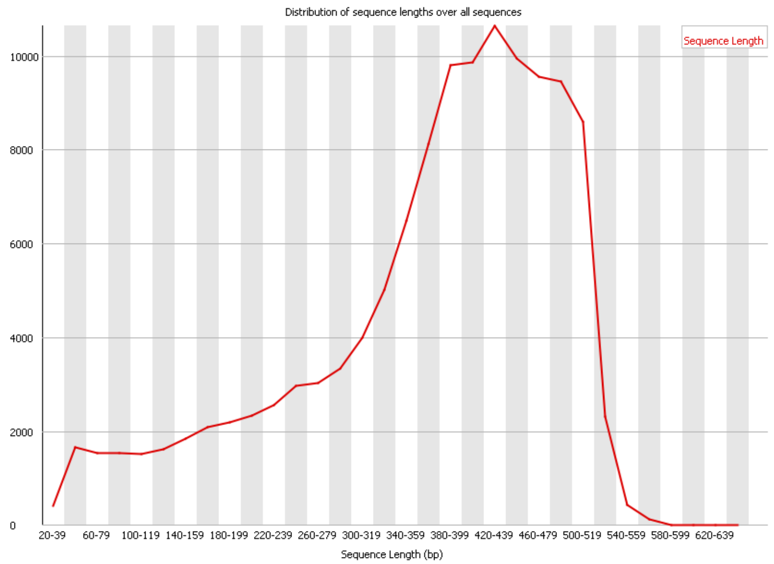

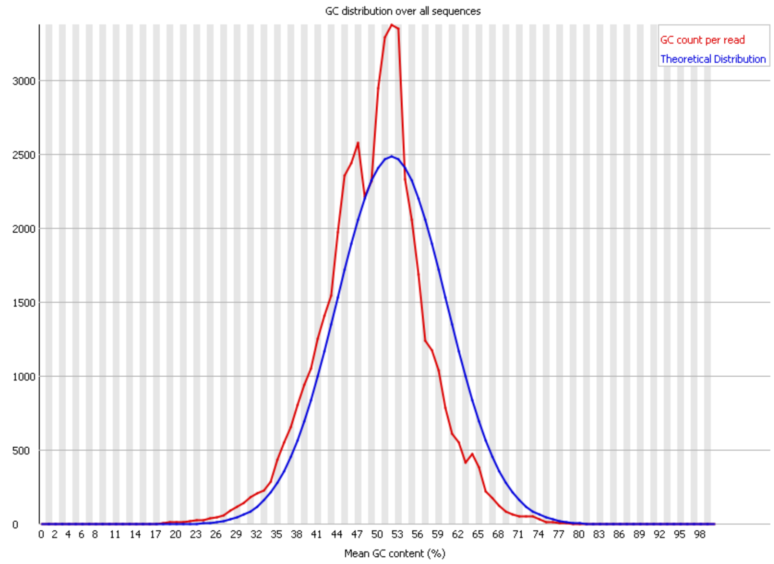

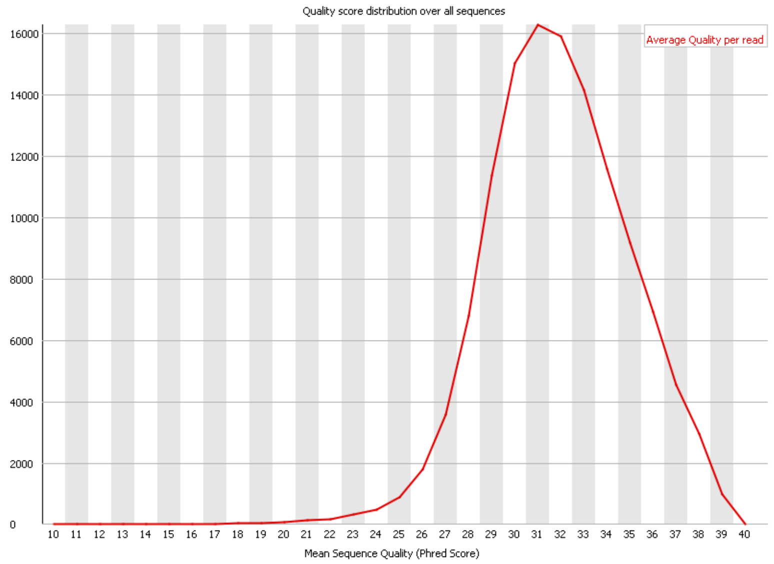

RNA-seq data quality assessment

2276 Resistant

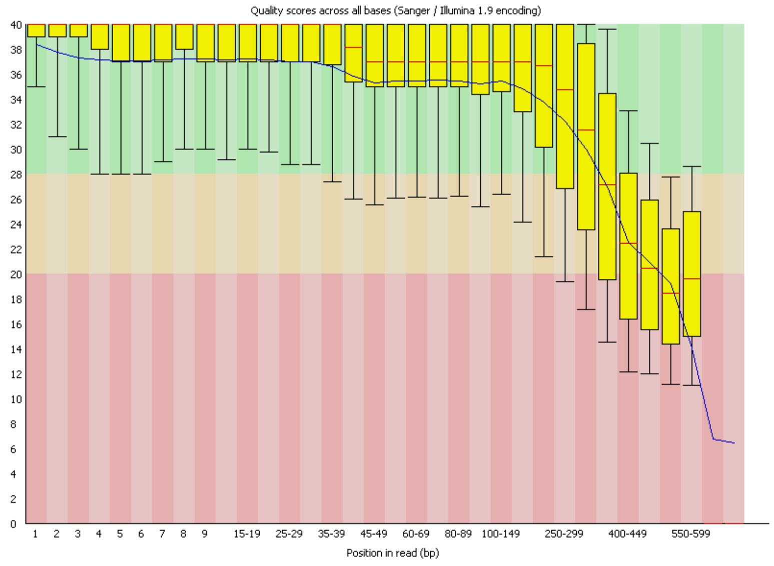

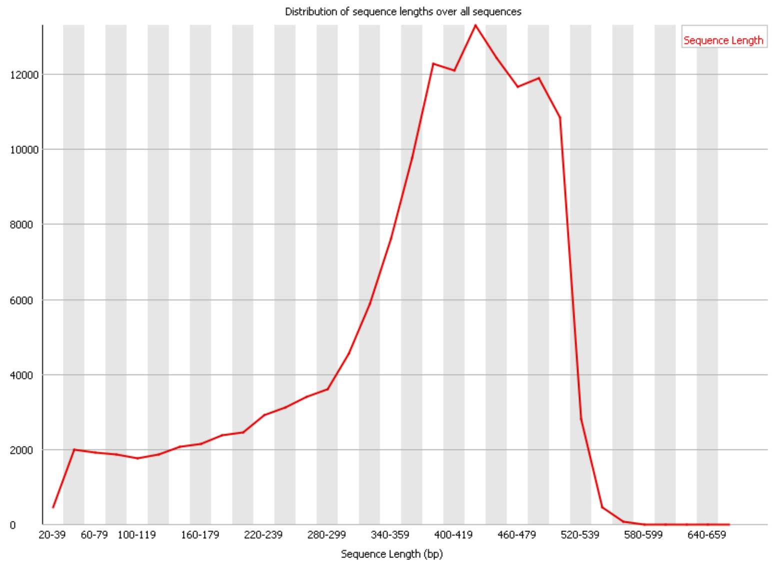

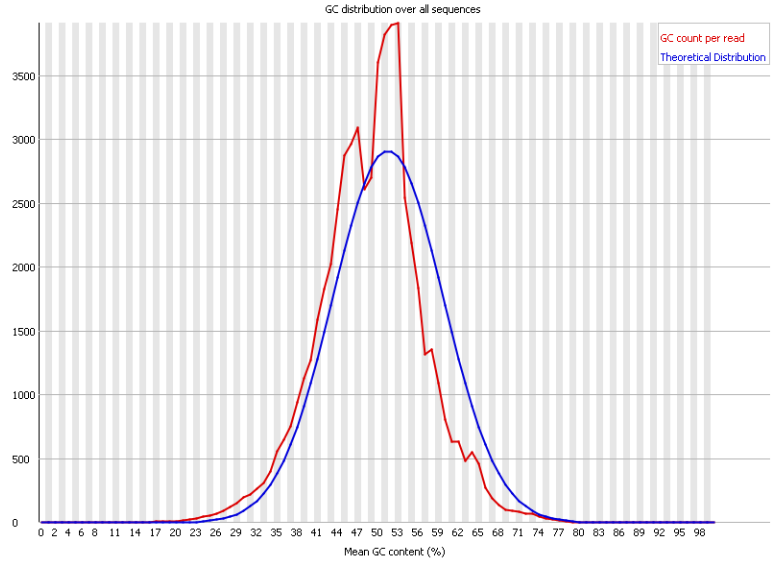

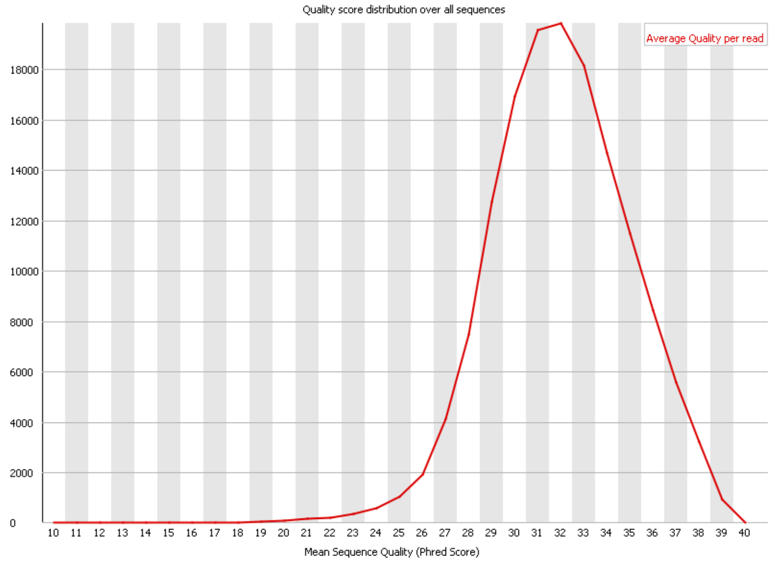

RNA-seq data quality assessment

1228 Resistant

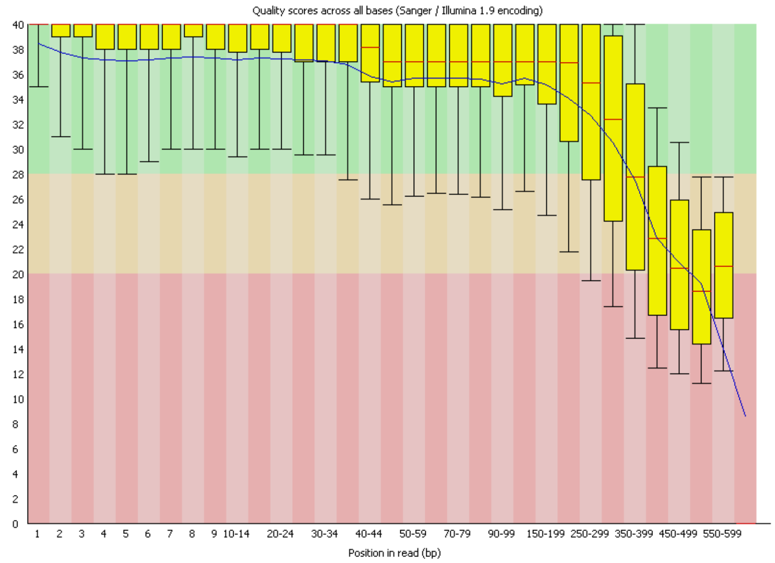

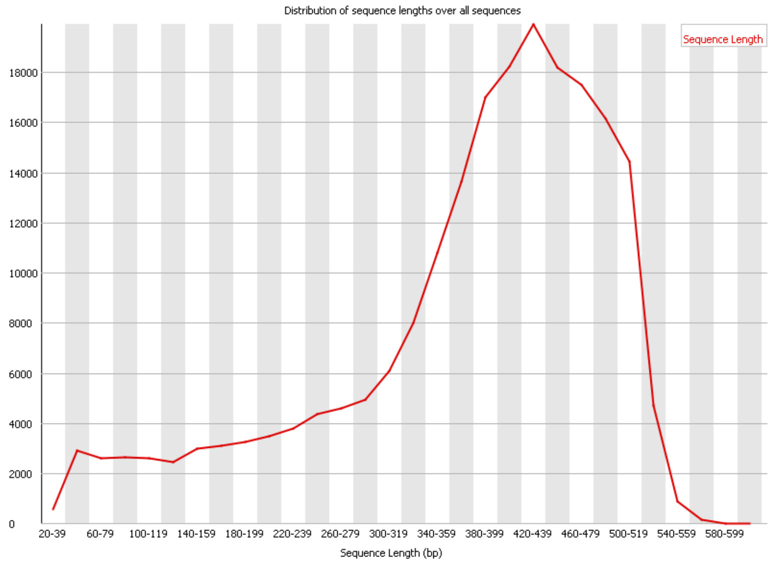

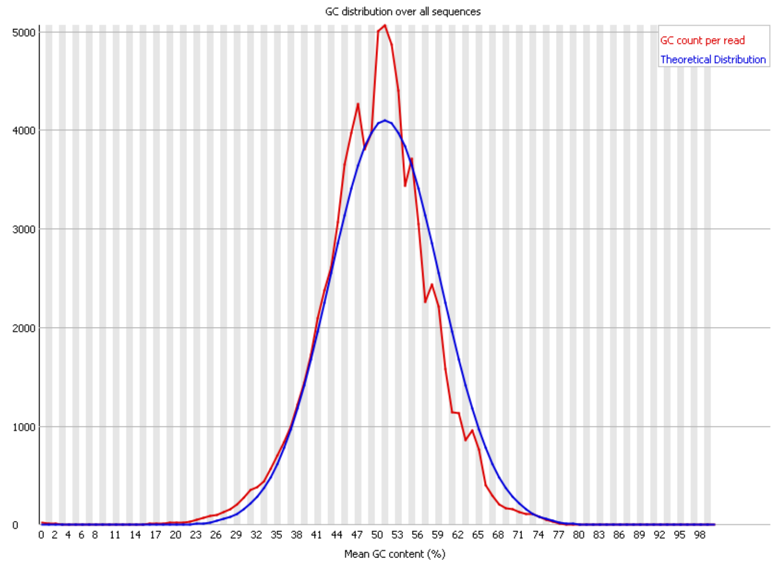

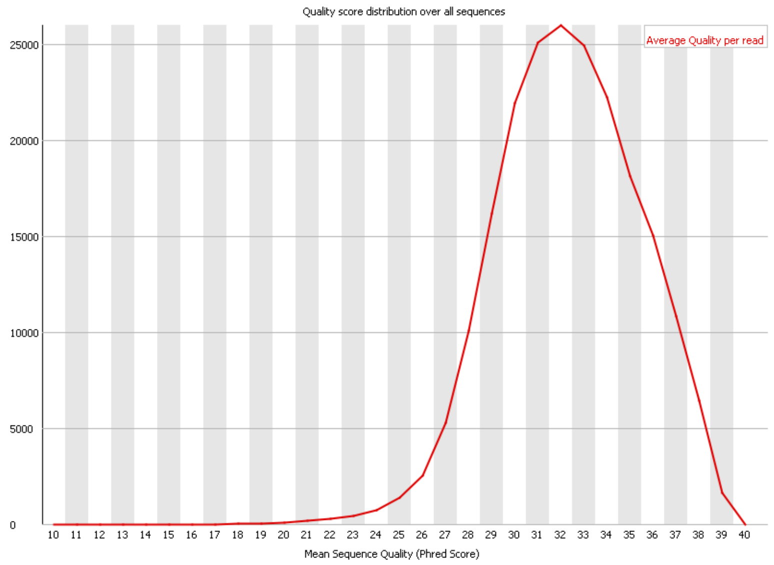

RNA-seq data quality assessment

2692 Resistant

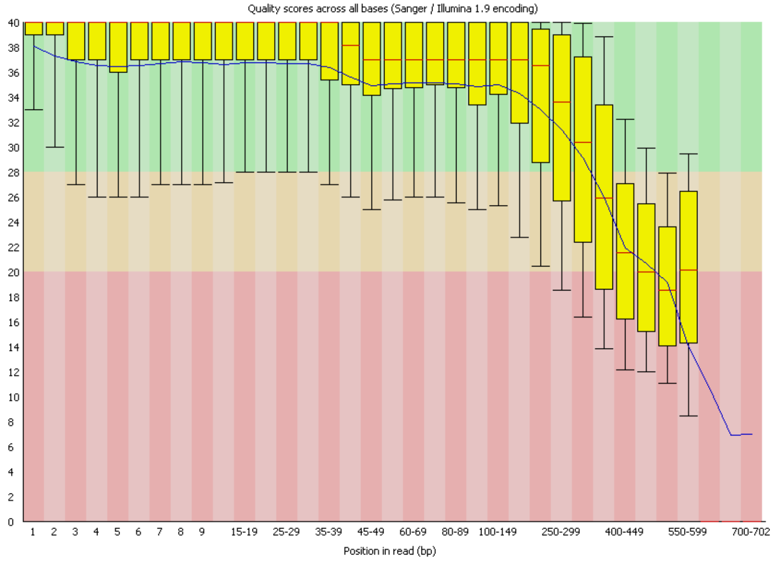

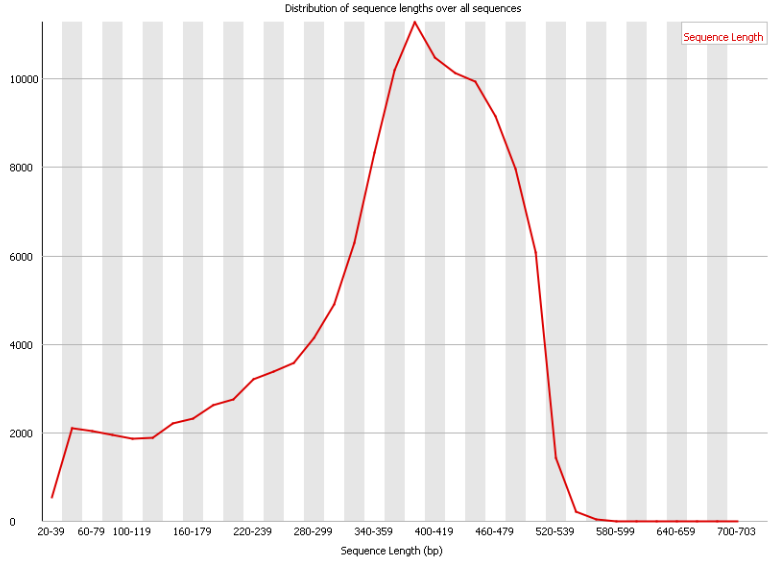

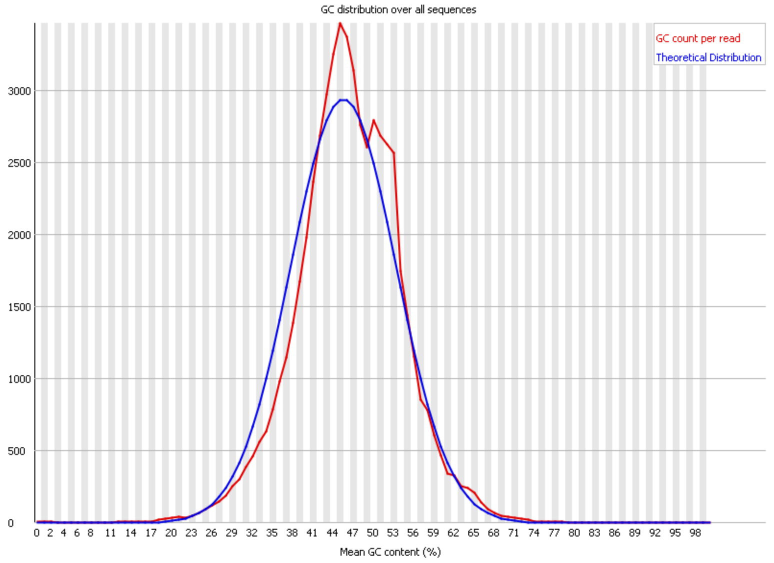

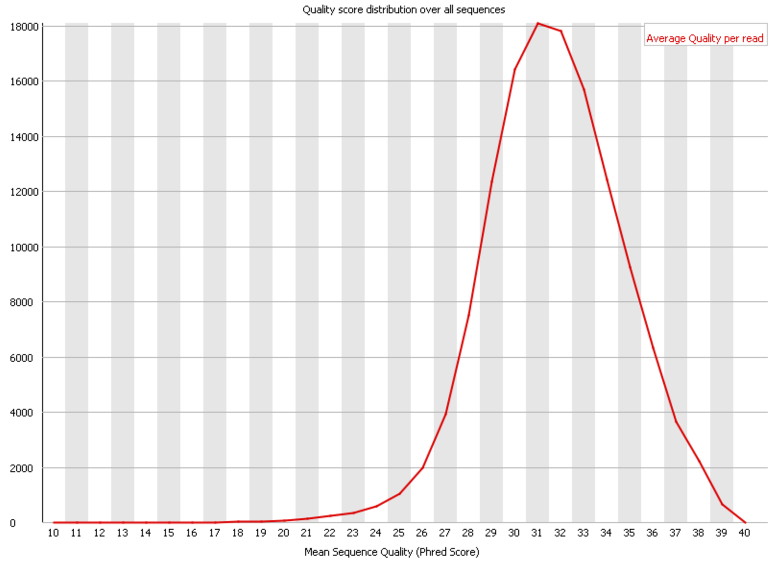

RNA-seq data quality assessment

2143 Susceptible

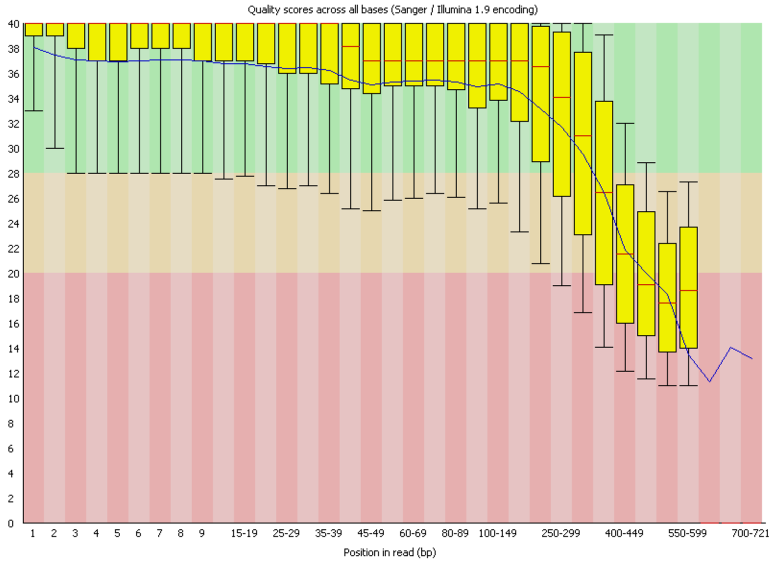

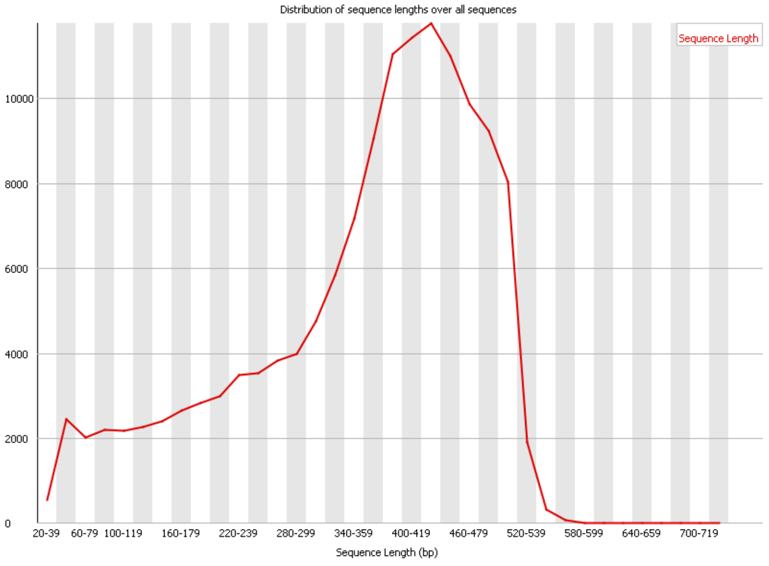

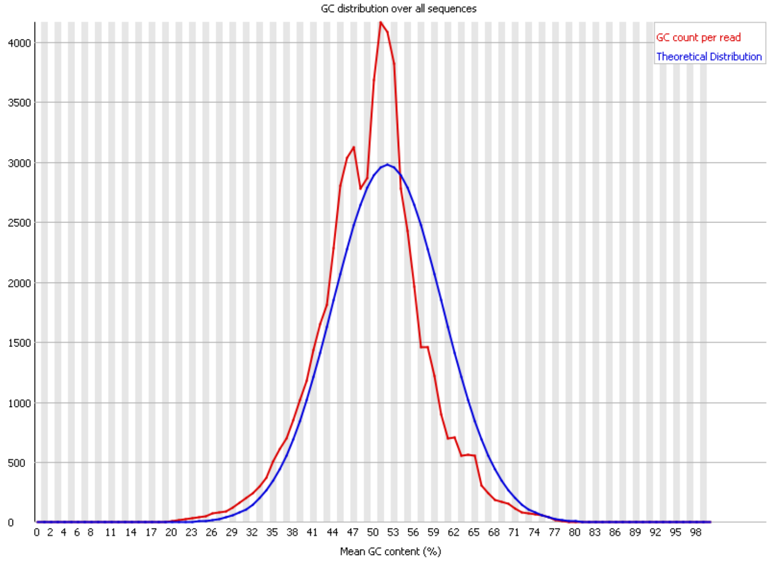

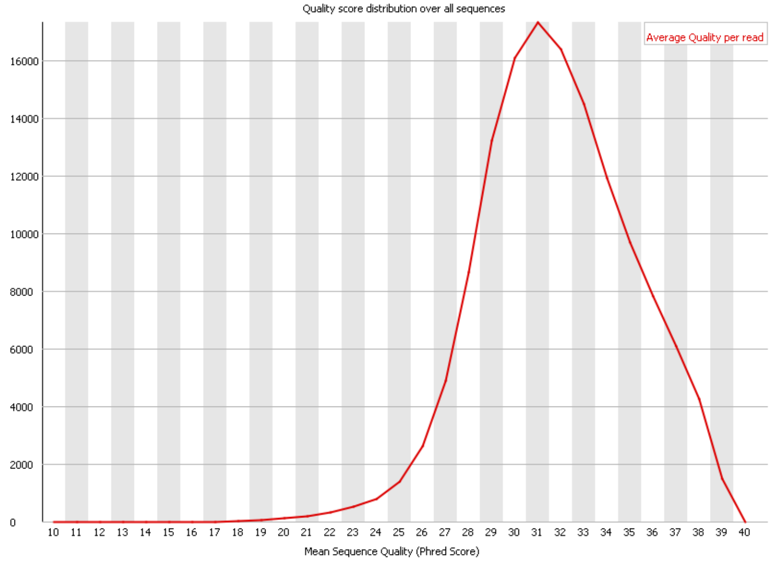

RNA-seq data quality assessment

1973 Susceptible

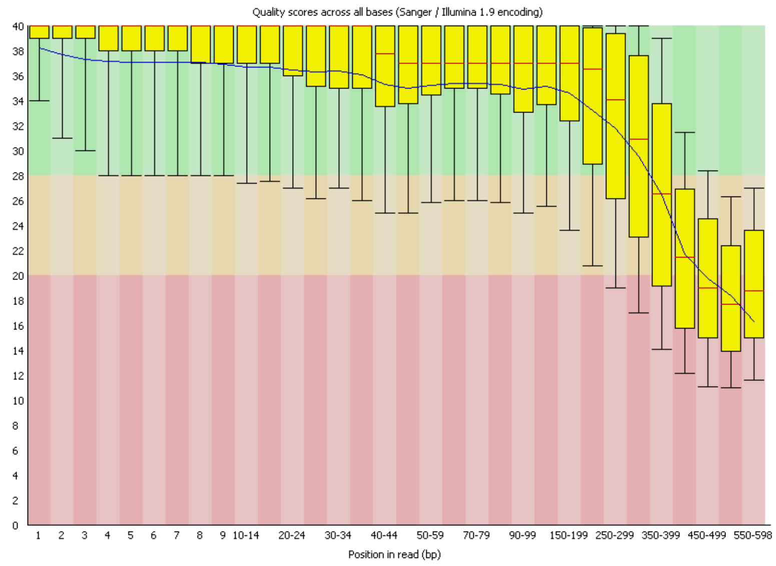

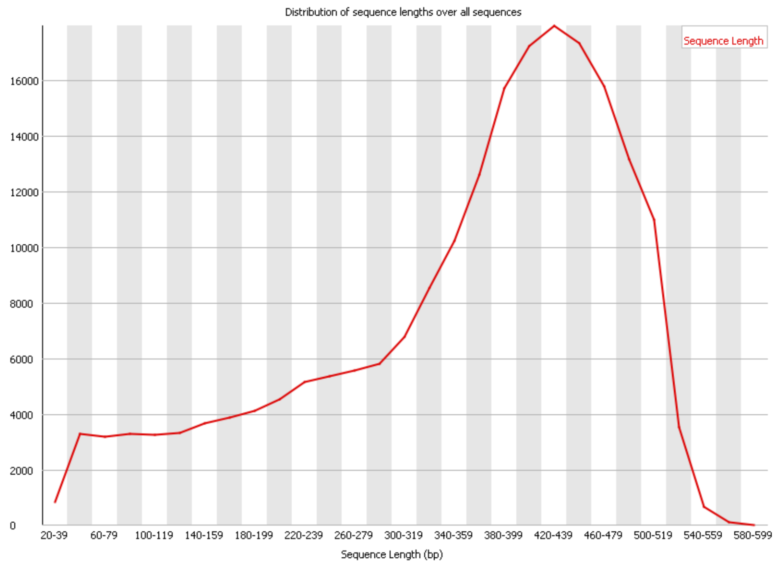

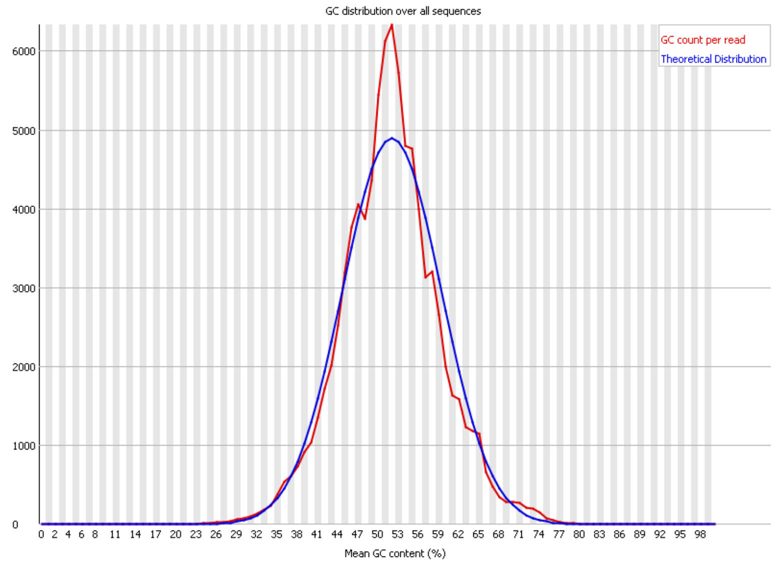

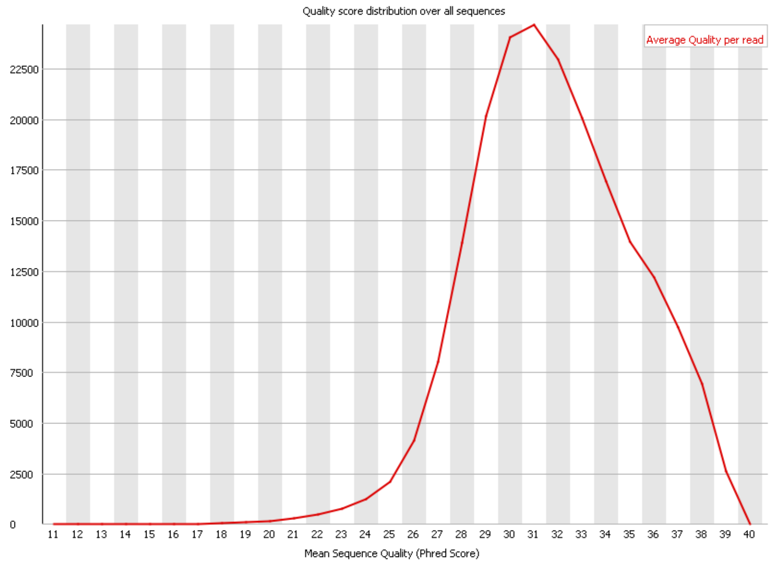

RNA-seq data quality assessment

3128 Susceptible

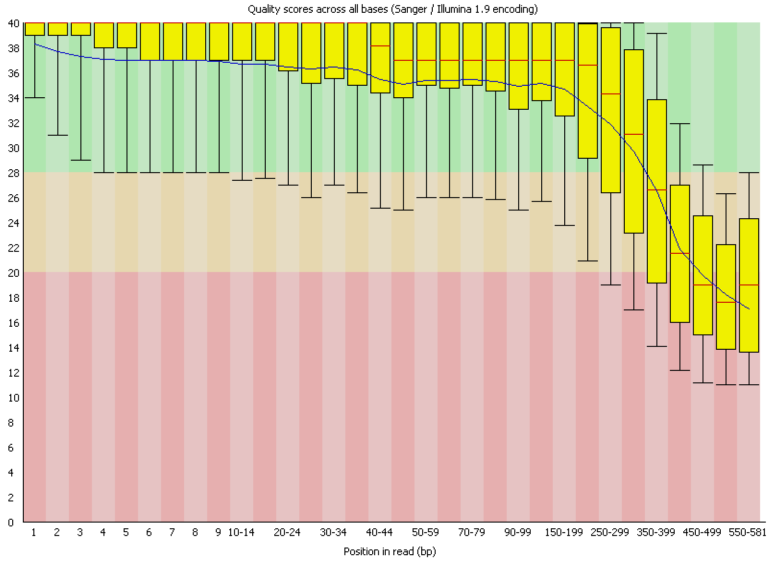

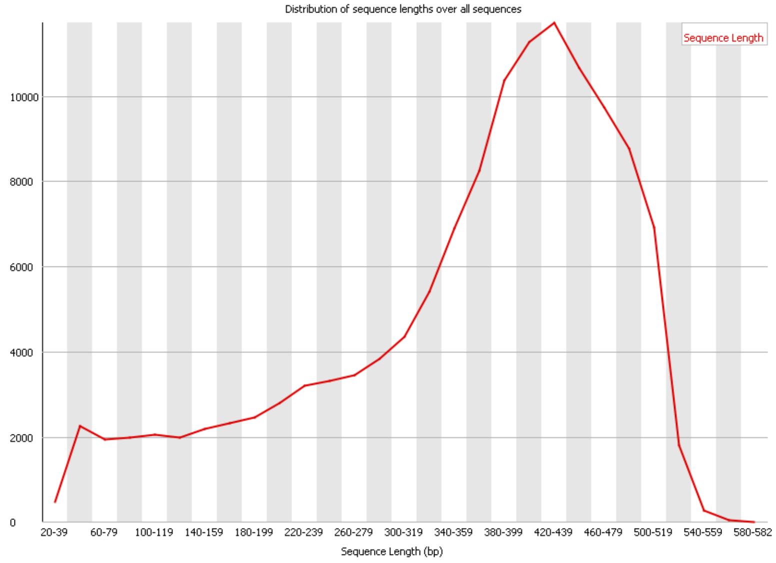

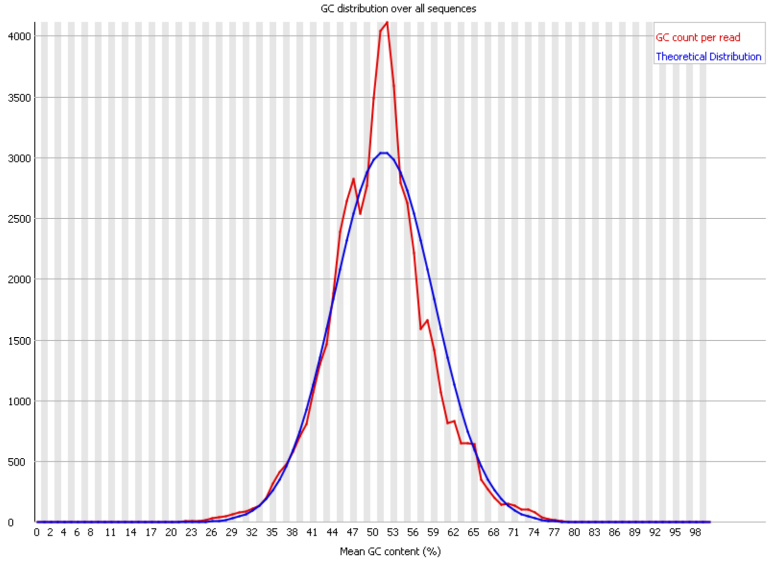

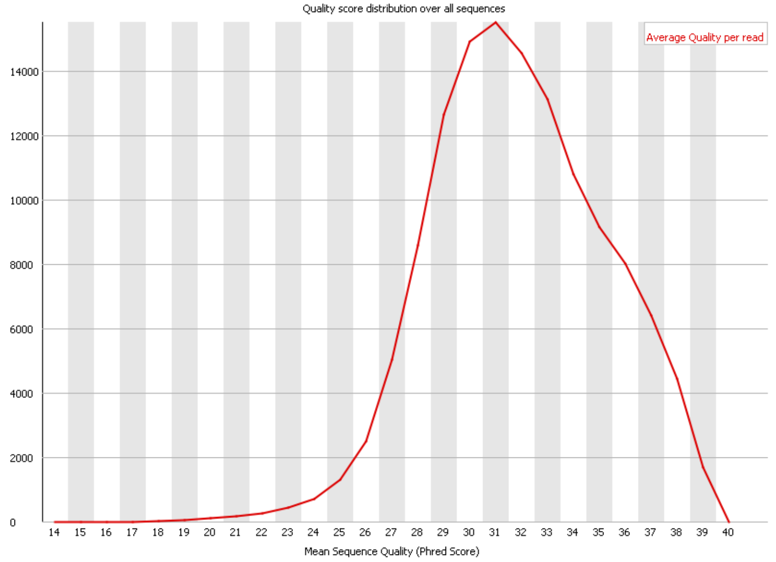

RNA-seq data quality assessment

DN00726 Susceptible

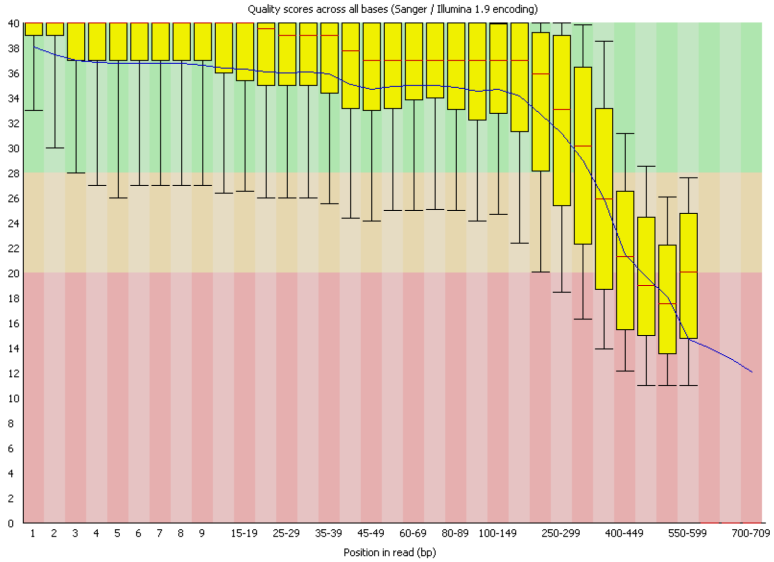

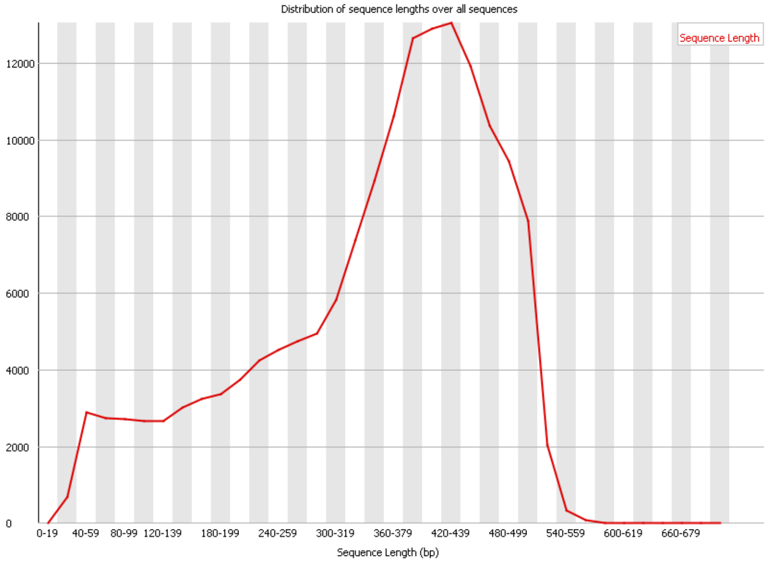

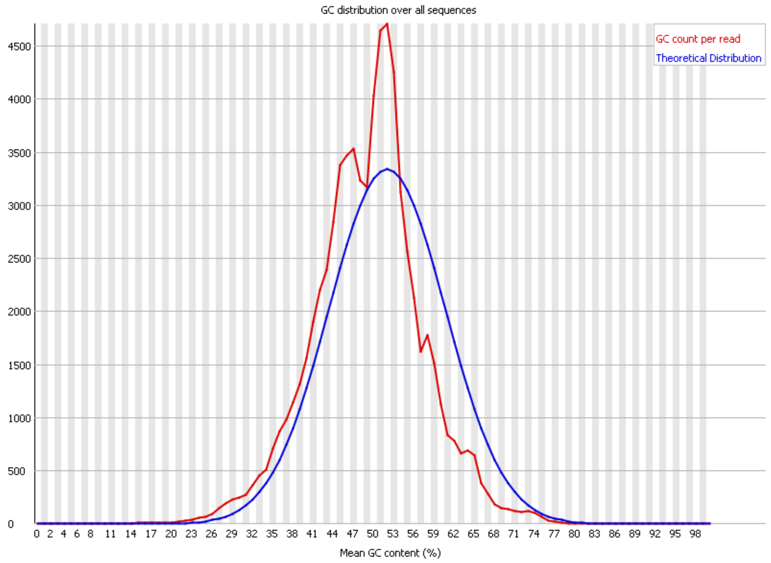

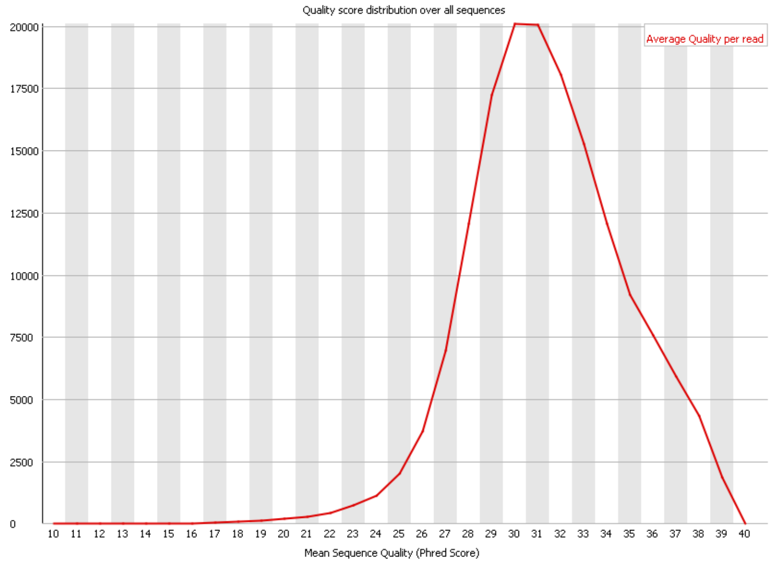

RNA-seq data quality assessment

Holden Susceptible

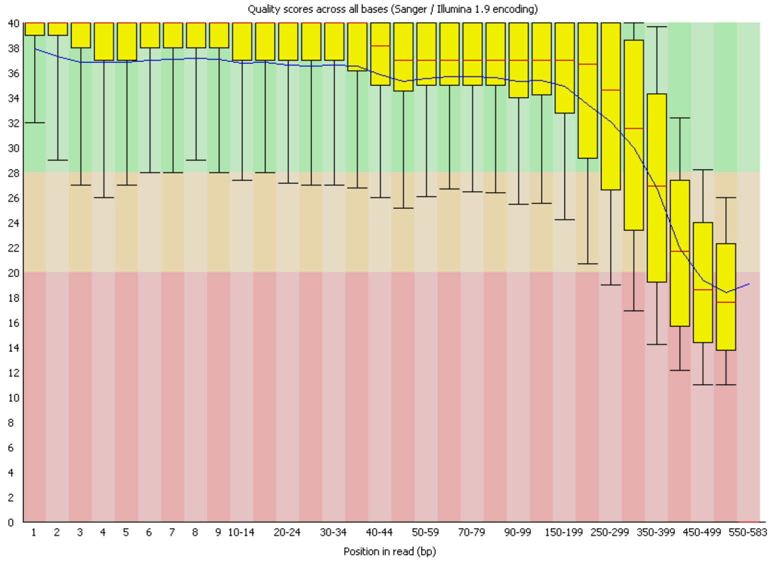

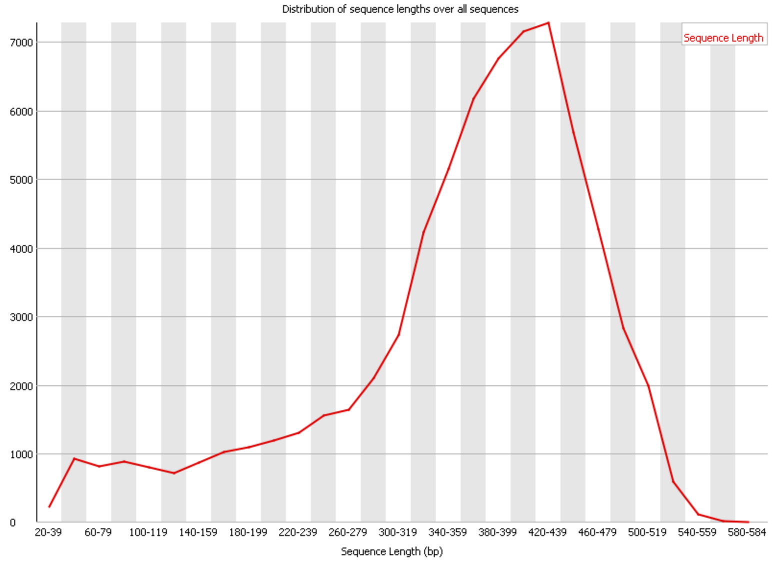

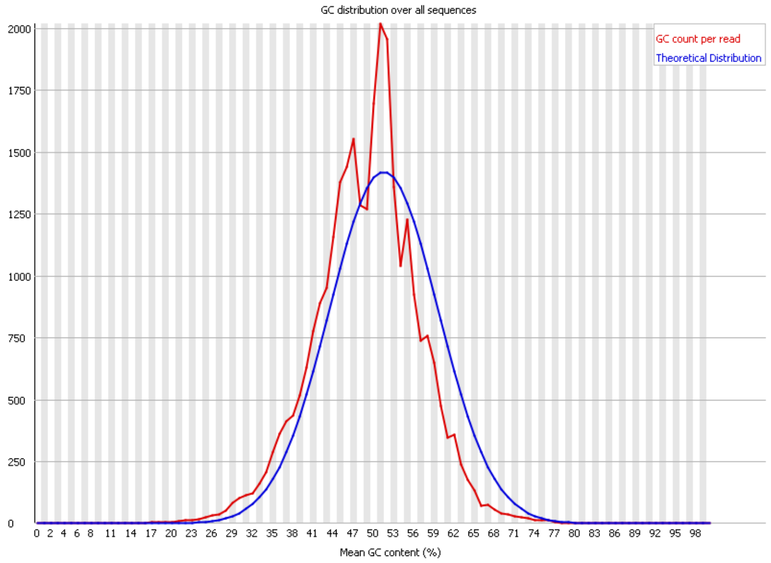

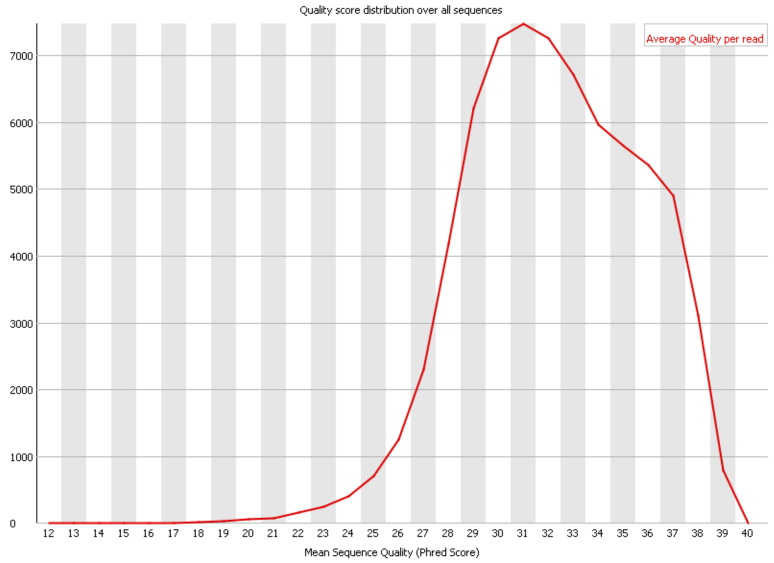

Supplement: Supplementary file 2 — RNA‐seq data quality assessment. (PDF 853 kb) [file 12864_2017_3931_MOESM2_ESM.pdf]
