# Supplementary material for: Genome-wide association study identifies a major gene for beech bark disease resistance in American beech (Fagus grandifolia Ehrh.)
Source: BMC Genomics. 2017 Jul 20;18:547. doi: 10.1186/s12864-017-3931-z (PMC5520234; doi:10.1186/s12864-017-3931-z)

Examples RNA vs mRNA Agilent Bioanalyzer quality profiles

Total RNA bioanalyzer profiles for samples 1228R and 2143S

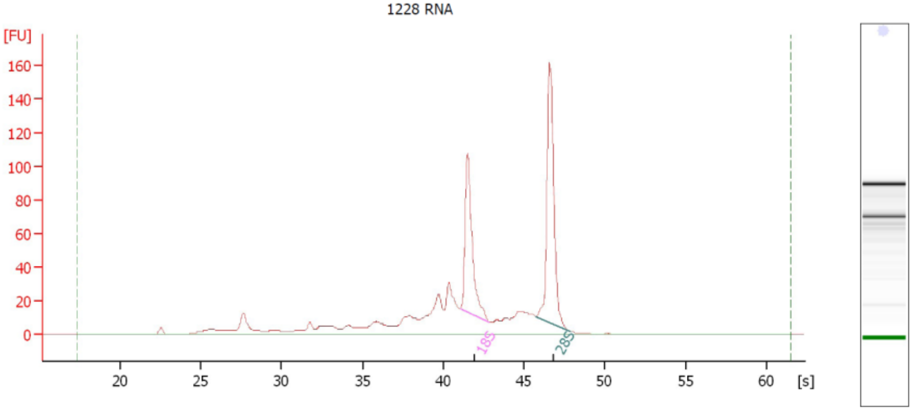

Overall Results for sample 1 : 1228 RNA

RNA Area:	735.8	RNA Integrity Number (RIN):	7.7 (B.02.07)
RNA Concentration:	1,443 ng/µl	Result Flagging Color:	
rRNA Ratio [28s / 18s]:	1.3	Result Flagging Label:	RIN: 7.70

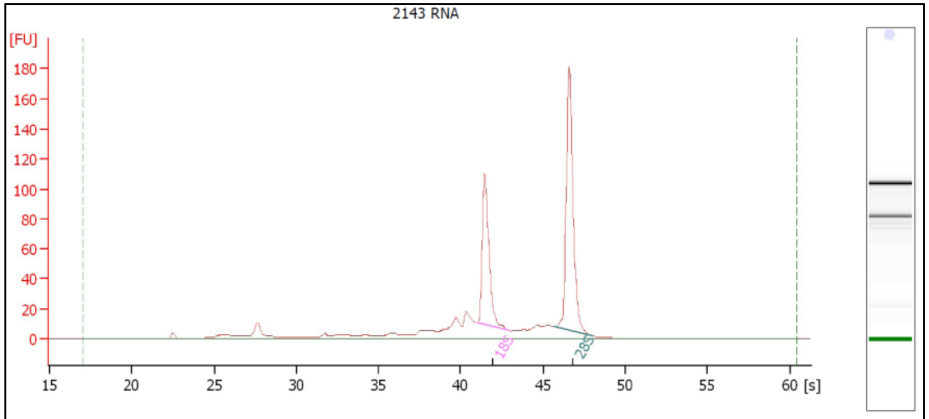

Overall Results for sample 2 : 2143 RNA

RNA Area:	585.9	RNA Integrity Number (RIN):	8.5 (B.02.07)
RNA Concentration:	1,149 ng/µl	Result Flagging Color:	
rRNA Ratio [28s / 18s]:	1.6	Result Flagging Label:	RIN: 8.50

mRNA bioanalyzer profiles for samples 1228R and 2143S

1st Poly-A Elution (100 ul)

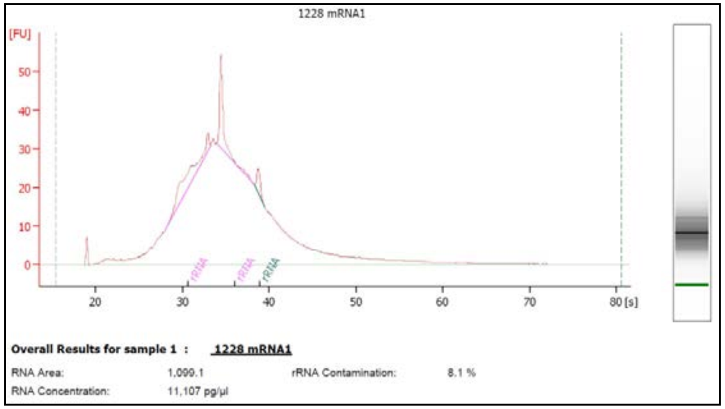

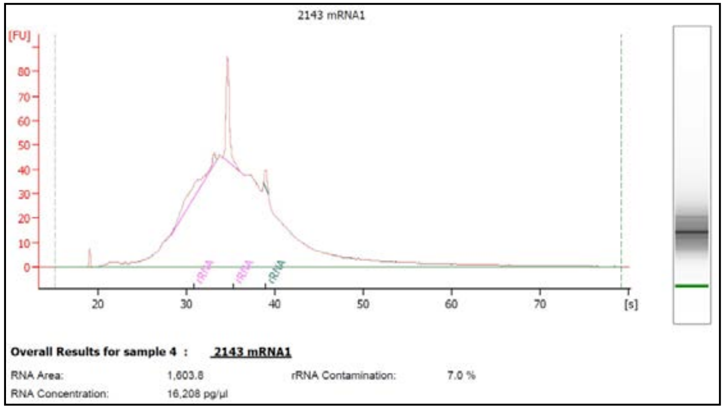

2nd Poly-A Elution (100 ul)

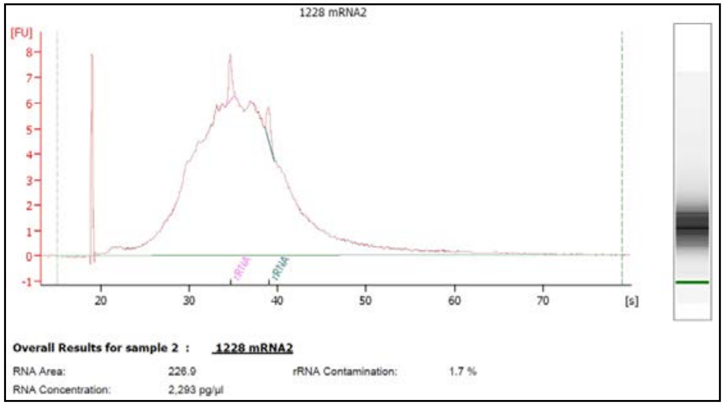

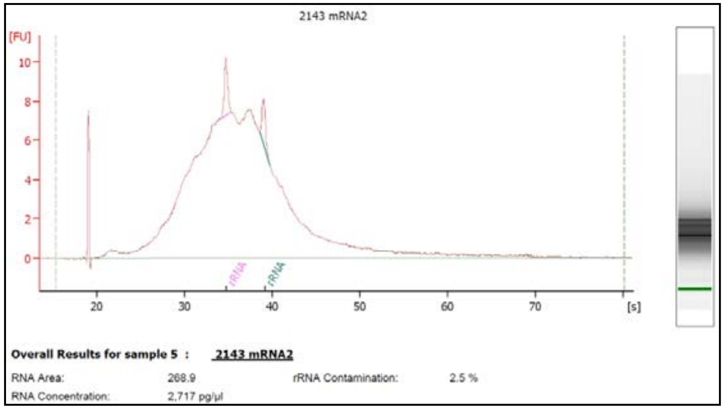

Poly-A 3rd Elution (100 ul)

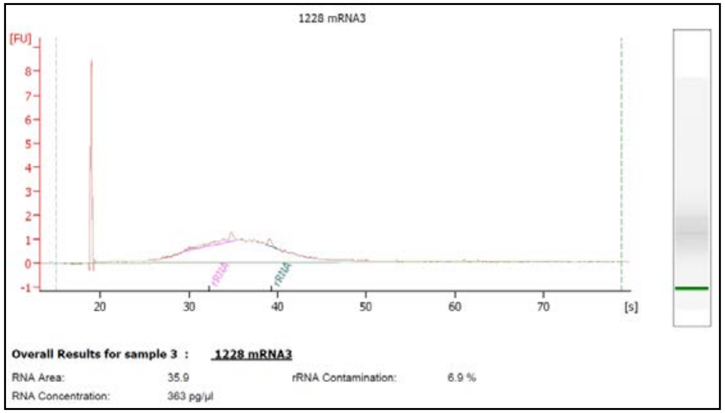

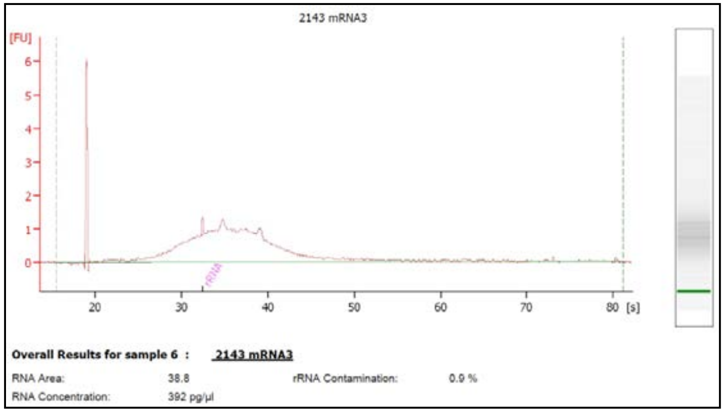

Supplement: Supplementary file 3 — Examples of RNA vs. mRNA Agilent Bioanalyzer quality profiles. (PDF 288 kb) [file 12864_2017_3931_MOESM3_ESM.pdf]
