# Supplementary material for: Genome-wide association study identifies a major gene for beech bark disease resistance in American beech (Fagus grandifolia Ehrh.)
Source: BMC Genomics. 2017 Jul 20;18:547. doi: 10.1186/s12864-017-3931-z (PMC5520234; doi:10.1186/s12864-017-3931-z)

Additional File 6 (A) DAPC analysis revealed three main genetic clusters where the individuals shown as dots and the groups as inertia ellipses. Eigenvalues of the analysis are displayed inset. (B) Quantile-quantile (QQ) plot of GWA p-values where on x axis, are expected $–\log_{10} P$ values and on y axis observed $–\log_{10} P$ values. The plot is showing large deviation from the null distribution where the inflation factor was higher than the threshold of 1, indicating a high genomic inflation in Beech association data and an existence of the population stratification.

A

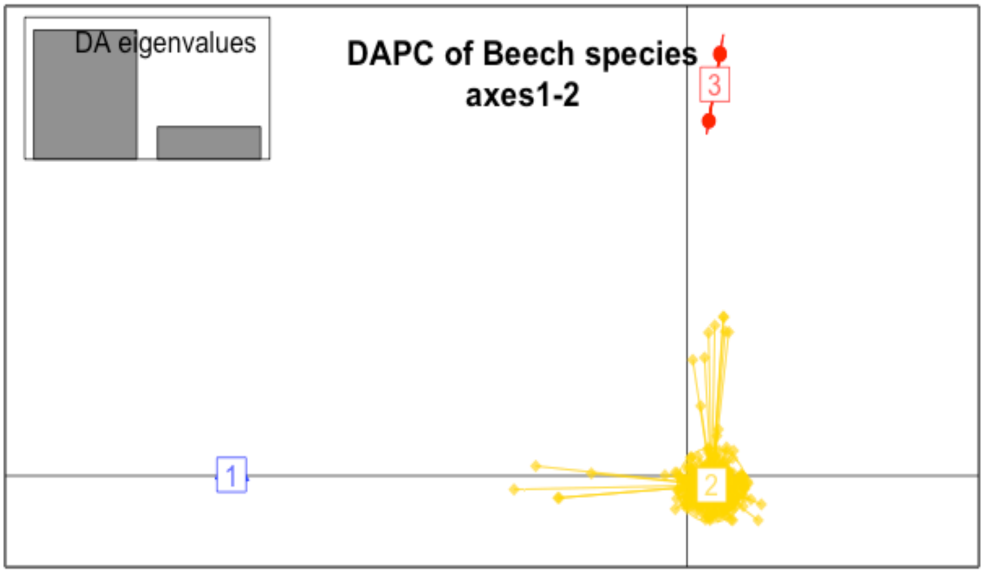

B

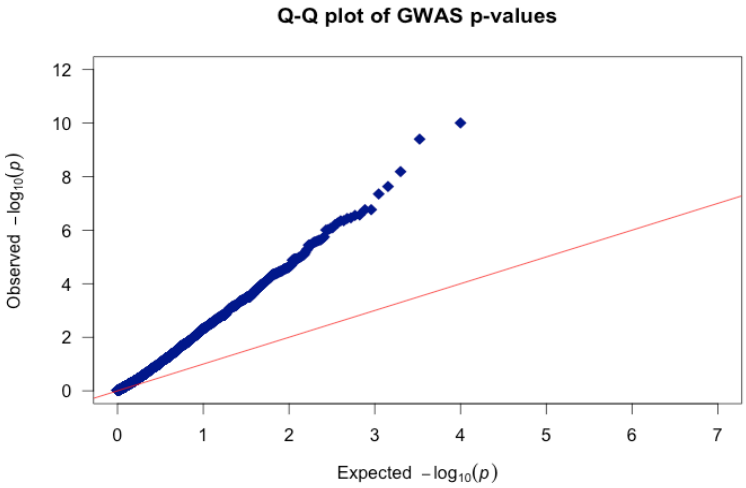

Supplement: Supplementary file 6 — (A) DAPC analysis revealed three main genetic clusters where the individuals shown as dots and the groups as inertia ellipses. Eigenvalues of the analyses are displayed inset. (B) Quantile-quantile (QQ) plot of GWA p-values where on x-axis, are expected –log10 P values and on y-axis observed –log10 P values. The plot is showing large deviation from the null distribution where the inflation factor was higher than the threshold of 1, indicating a high genomic inflation in Beech association data and an existence of the population stratification. (PDF 90 kb) [file 12864_2017_3931_MOESM6_ESM.pdf]
