# Supplementary material for: Genome-wide association study identifies a major gene for beech bark disease resistance in American beech (Fagus grandifolia Ehrh.)
Source: BMC Genomics. 2017 Jul 20;18:547. doi: 10.1186/s12864-017-3931-z (PMC5520234; doi:10.1186/s12864-017-3931-z)
Supplement: Supplementary file 8 — (A) Four highlighted markers with significance level higher than genome-wide threshold (P value >1.585 × 10–﻿﻿5﻿) located on the chromosome (Chr) 5. (B) Alignment of four nucleotide sequences to reference sequence Fagus sylvatica mRNA (Sequence ID: AJ130886.1). The FASTA sequence order corresponds as AX-156994126 (SEQ_1), AX-156989406 (SEQ_2), AX-156988334 (SEQ_3) and AX-157000652 (SEQ_4). Highlighted red nucleotides refer to polymorphism to reference sequence and green nucleotides present diagnostics SNPs, respectively. (DOCX 4155 kb) [file 12864_2017_3931_MOESM8_ESM.docx]

Additional File 8. (A) Four highlighted markers with significance level higher than genome-wide threshold (P value > 1.585 x 10^-4^) located on the chromosome (Chr) 5.


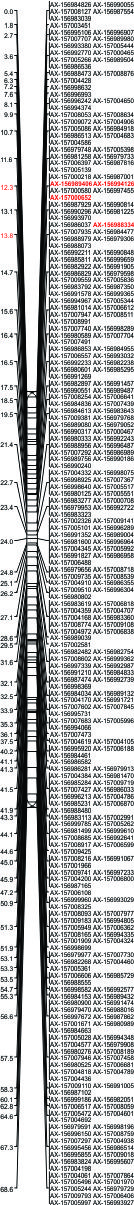


(B) Alignment of four nucleotide sequences to reference sequence *Fagus sylvatica* mRNA (Sequence ID: AJ130886.1). The FASTA sequence order corresponds as AX-156994126 (SEQ_1), AX-156989406 (SEQ_2), AX-156988334 (SEQ_3) and AX-157000652 (SEQ_4). Highlighted red nucleotides refer to polymorphism to reference sequence and green nucleotides present diagnostics SNPs, respectively.

| 1-70bp |  |
| --- | --- |
| Ref_seq | CCAAAATCCAGTTTTTGCATCAATCTTTAGCTCTTGAAAAATGTCGTGCTGCGGAGGAAACTGTGGATGT |
| SEQ_1 | ______________________________________________________________________ |
| SEQ_2 | ______________________________________________________________________ |
| SEQ_3 | ______________________________________________________________________ |
| SEQ_4 | ________________________________CTTGAAAAaTGTCGTGCTGCGGAGGAAACTGTGGATGT |
| 71-140bp |  |
| Ref_seq | GGTACTGGCTGCAAGTGCGGCAGCGGCTGTGGAGGATGCAAGGCGTACCCTGACTTGAGCTACACCGAGA |
| SEQ_1 | ______________________________________________________________________ |
| SEQ_2 | ______________________________________________________________________ |
| SEQ_3 | ______________________________________________________________________ |
| SEQ_4 | GGTACTGGCTGCAAGTGCGGCAGCGGCTGTGGAGGATGCAAGGCGTACCCTGACTTGAGCTA**Y**ACCGAGA |
| 141-210bp |  |
| Ref_seq | AGACCACCACTGAGACCCTTATTGTTGGTGTTGCTCCCCAGAAGGCACACTCTGAGGGATCTGAGATGGG |
| SEQ_! | ______________________________________________________________________ |
| SEQ_2 | ______________________________________________________________________ |
| SEQ_3 | ________________________________________________________GGATCTGAGATGGG |
| SEQ_4 | AGACCACCACTGAGACCCTTATTGTTGGTGTTGCTCCCCAGAAGGCACACTCcGAGGGATCTGAGATGGG |
| 211-280bp |  |
| Ref_seq | CGTTGGAGCTGAGAATGGGGGCTGCAAGTGTGGATCCAACTGCACCTGTGACCCTTGCAATTGTAAATGA |
| SEQ_1 | ______________________________________________________________________ |
| SEQ_2 | ______________________________________________________________________ |
| SEQ_3 | CGTTGGAGCTGAGAATGGAGGCTGCAAGTGTGGATCCAACTGCACCTGTGACCCTTGCAATTGTAAATGA |
| SEQ_4 | CGTTGGAGCTGAGAATGGAGGCT |
| 281-350bp |  |
| Ref_seq | GAGGGATGCTAGCCTTTCAAGCAGAGACAAGGAATTTAATAAATACTAGCTTGTAGTATTGTTGTATATG |
| SEQ_1 | ___________________________CAAGGAATTTAATAAaTACTAGCTTGTAGTATTGTTGTATATG |
| SEQ_2 | ______________________________________________________________________ |
| SEQ_3 | GAGGGATGCTAGCCtT**Y**CAAGCAGAGACAAGGAATTTAATAAaTACTAGCTTGTAGTATTGTTGTATATG |
| 351-420bp |  |
| Ref_seq | TGTTGTGTCTAAGACTCATGACGTGTTCCTGAGAACTCATTTAATTTAAAATAAGGTGATCATGATTGTC |
| SEQ_1 | TGTTGTGTCTAAGACTCATGACGTGTTCCTGAGAACTCCTTTAATTTAAAATAAGGT**S**ATCATGATTGTC |
| SEQ_2 | ______________________________________________________________________ |
| SEQ_3 | TGTTGTGTCTAAGACTCATGACGTGTTCCTGAGAACTCCTTTAATTT |
| 421-490bp |  |
| Ref_seq | TGTATGCAAGTCTTACTAGTCTAAGGTTTGCCTTGGGTTCCCTCGAAGAACCATGGTTGCTTGGTTTATG |
| SEQ_1 | TATATGCAAGTCTTACTAGTCTAAGGTTTGCCTTGGGTTCCCTtXXXXXXXXXXGGTTGCTTGGTTTATG |
| SEQ_2 | ______________________AAGGTTTGCCTTGGGTTCCCTtXXXXXXXXXXGGTTGCTTGGTTTATG |
|  |  |
| 491-560bp |  |
| Ref_seq | TTTTCTTTGTGCTATGGTTGTAGTAAGTTATGTATGATGGGAATGGGAGTCTCTGCTGTAATGGGATAAT |
| SEQ_1 | TTTTCTTTGTGCTATGGTTGTAGTAAGT |
| SEQ_2 | TTTTCTTTGTGCTATGGTTGTAGTAAGTTATGTATGATGGGAAT**K**GGAGTCTCTGCTGTAATGGgATAAT |
|  |  |
|  |  |
| 561-630bp |  |
| Ref_seq | ATATGTGGGAAGAGAGTTTTAATATTCGAATGAAAACTCCTTTCTTCTAAAAAAAAAAAAAAAAAAAA |
| SEQ_1 |  |
| SEQ_2 | ATATGTGGGAAGAGAGTTTTAATATTCGAATGAAAACTCCTTTCTTCTACTTCTAGCTTCAAATAACAGT |
|  |  |
|  |  |
|  |  |
|  |  |
|  |  |
|  |  |
|  |  |
