# Supplementary material for: Genome-wide association study identifies a major gene for beech bark disease resistance in American beech (Fagus grandifolia Ehrh.)
Source: BMC Genomics. 2017 Jul 20;18:547. doi: 10.1186/s12864-017-3931-z (PMC5520234; doi:10.1186/s12864-017-3931-z)

Additional File 9. RNA sequence reads from each cDNA library to the full-length copy of the candidate gene transcript sequence from contig 03321, representing the expression of the candidate *Mt* gene after the challenge by the insect vector.


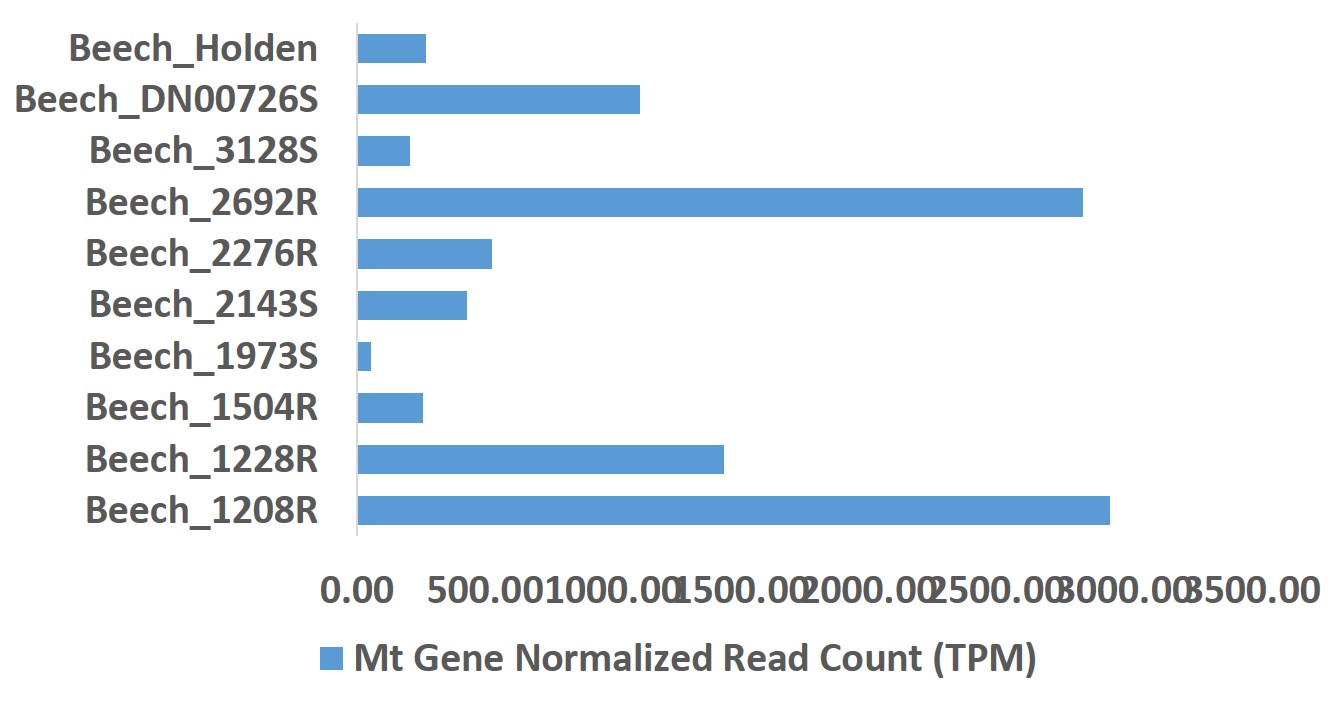

Supplement: Supplementary file 9 — RNA sequence reads from each cDNA library mapped to the full-length copy of the candidate gene transcript sequence from contig 03321, representing the expression of the candidate Mt gene after the challenge by the insect vector. (DOCX 161 kb) [file 12864_2017_3931_MOESM9_ESM.docx]
