# Supplementary material for: Genome-wide association study identifies a major gene for beech bark disease resistance in American beech (Fagus grandifolia Ehrh.)
Source: BMC Genomics. 2017 Jul 20;18:547. doi: 10.1186/s12864-017-3931-z (PMC5520234; doi:10.1186/s12864-017-3931-z)

Additional File 10 Linkage disequilibrium (LD) structure across four SNPs associated to BBD. (Red) strong LD between markers; (white) no LD. The block-like pattern represents the regions of high LD. Pairwise LD among four SNPs listed as squared allelic correlation (r^2^) (Pritchard and Przeworski 2001) and Lewontin’s D’ (Lewontin 1964).

1. AX-157000651, AX-156988334 D’:1.0; LOD:81.34; r-squared:0.985
2. AX-156988334, AX-156994126 D’:1.0; LOD:78.3; r-squared:0.985
3. AX-156994126, AX-156989406 D’:1.0; LOD:78.0; r-squared:0.985
4. AX-157000652, AX-156994126 D’:1.0; LOD:76.89, r-squared:0.97
5. AX-156988334, AX-156989406 D’:0.985, LOD:75.26; r-squared:0.956
6. AX-157000652, AX-156989406 D’:0.985, LOD:74.07, r-squared:0.942


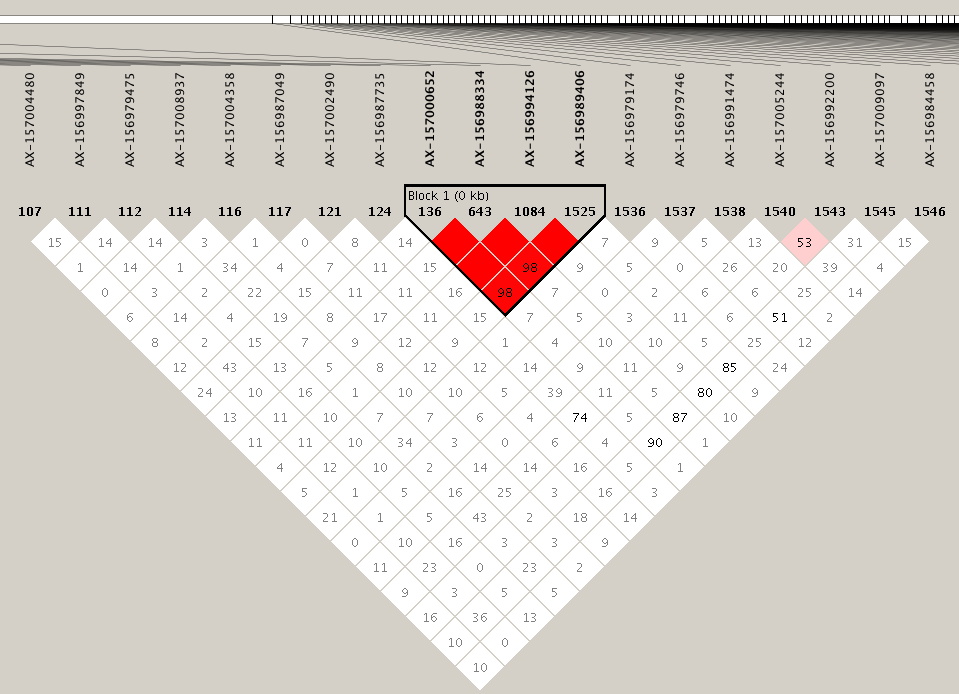

Supplement: Supplementary file 10 — Linkage disequilibrium (LD) structure across four SNPs associated to BBD. (Red) strong LD between markers; (white) no LD. The block-like pattern represents the regions of high LD. Pairwise LD among four SNPs listed as squared allelic correlation (r2) [61] and Lewontin’s D’ [62]. (DOCX 162 kb) [file 12864_2017_3931_MOESM10_ESM.docx]
